# Auto‐Downregulation of the Florigen FT Production Prevents Precocious Flowering in Plants

**DOI:** 10.1002/advs.202522307

**Published:** 2026-07-13

**Authors:** Shu Tian, Xiao Luo, Bowen Cui, Yuehui He

**Affiliations:** ^1^ Peking‐Tsinghua Center for Life Sciences & State Key Laboratory of Wheat Improvement School of Advanced Agricultural Sciences Peking University Beijing China; ^2^ College of Agronomy Shandong Agricultural University Tai'an Shandong China; ^3^ Shandong Laboratory of Advanced Agricultural Sciences in Weifang Peking University Institute of Advanced Agricultural Sciences Shandong China

**Keywords:** auto‐downregulation, FD, florigen, FT, photoperiodic flowering

## Abstract

In many plants, flowering is timed by seasonal changes in the length of daylight (photoperiod). In *Arabidopsis thaliana*, long‐day exposure results in an increasing buildup of the CONSTANS (CO) protein toward the end of daylight, which activates the expression of the major florigen gene *FLOWERING LOCUS T* (*FT*) to induce flowering. CO‐mediated *FT* activation must be properly controlled to prevent an excessive florigen production and precocious flowering under inductive photoperiods, but the underlying mechanism remains elusive. Here, we report an auto‐repression mechanism to prevent excessive FT production in inductive photoperiods. We show that the transcription factor FD is expressed in leaf veins and complexes with FT to recognize several *cis*‐regulatory DNA motifs in *FT* promoter. FT‐FD antagonizes CO‐mediated *FT* activation to feedback down‐regulate *FT* expression and thus prevent its excessive induction by long‐day signals, thereby precluding precocious transition to flowering. Furthermore, we found that in the facultative short‐day plant soybean, an *FT* homolog directly represses its own expression. Thus, the auto‐repression of *FT* or an *FT* homolog is a conserved mechanism to prevent excessive production of this potent floral regulator, ensuring the floral transition at a proper time to balance vegetative growth with reproductive success and maximize plant production.

## Graphical abstract

This study shows that the leaf vein‐expressed FD complexes with the florigen FT to bind DNA motifs in *FT* promoter, which antagonizes CONSTANS‐mediated *FT* activation to feedback down‐regulate *FT* expression. This prevents an excessive induction of *FT* expression by inductive photoperiods, thereby precluding precocious transition to flowering in Arabidopsis. The auto‐repression of *FT* or *FT* homologs is a conserved mechanism in flowering plants.

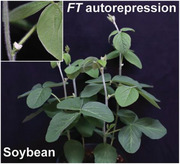

## 1 Introduction

In many flowering plants, the developmental transition to flowering or switch from vegetative growth to reproduction is timed by seasonal changes of day length through the photoperiod pathway, to achieve reproductive success under favorable seasons [1, 2]. Light signals are perceived by photoreceptors such as phytochromes and cryptochromes in leaves, and are further integrated with the circadian clock to generate rhythmic expression of a transcriptional activator or regulator for florigen production [1, 2]. This leads to floral induction under inductive photoperiods.

In the facultative long‐day plant *Arabidopsis thaliana*, the *CO‐FT* regulatory module determines the photoperiodic flowering response. Under long day (LD) conditions, the circadian‐clock regulation of the transcriptional activator *CO* results in a high‐level expression from late afternoon into night [3, 4]. Furthermore, the stability of CO protein is regulated post‐translationally. CO is stabilized by far‐red and blue light signals, but is destabilized by red light and two RING‐finger E3 ubiquitin ligases [2, 5]. The coincidence of high‐level of *CO* mRNA expression with the presence of both far‐red and blue light in late afternoon under LDs, results in the accumulation of CO protein toward the end of daytime (dusk) [4, 5]. CO functions to activate the expression of the florigen gene *FT*, the major output of the photoperiod pathway, conferring long‐day induction of flowering in Arabidopsis [1, 6].

The CO protein gradually accumulates in leaf veins from late afternoon toward dusk in LDs [5]. Upon that it reaches a threshold level with increasing light exposure, the light‐stabilized CO protein, together with two NUCLEAR FACTOR‐Y (NF‐Y) subunits B and C, constitute a trimeric transcriptional activator complex termed as CO‐NF [6, 7, 8]. This complex recognizes four *CO*‐responsive DNA elements (COREs) located in the *FT* proximal promoter to activate *FT* expression in LDs [9, 10]. Meanwhile, the distal enhancer bearing the CCAAT motif, recognized by a trimeric NF‐Y transcription factor complex, is brought close to the CORE region (bound by CO‐NF) by promoter looping, thereby activating *FT* expression [11, 12]. *FT* expression is gradually activated from late afternoon and peaks by the end of the day (light period) [3, 4]. Subsequently, rapid CO degradation by the ubiquitin‐proteasome system at night, leads to *FT* repression [5]. Hence, the accumulation of CO protein toward the end of daytime results in the rhythmic *FT* activation in LDs, enabling plants to align their timing of the transition to flowering with long‐day seasons.

FT, a homolog of phosphatidylethanolamine‐binding protein (PEBP), functions as a mobile signal and is transported from leaf veins where it is produced to the shoot apical meristem (SAM) through the phloem [13, 14, 15, 16]. In the SAM, the basic leucine zipper domain (bZIP) transcription factor FD complexes with a 14‐3‐3 protein and then the complex binds a target DNA motif; subsequently, the FT protein assembles with the DNA‐bound FD‐14‐3‐3 to form a transcriptional activation complex, termed as the florigen activation complex (FAC) [17, 18, 19, 20]. FAC directly activates the expression of the floral meristem identity genes in the SAM, including *APETALA1*, *LEAFY* and *CAULIFLOWER* [17, 18, 19]. FD has been reported to be expressed preferentially in the SAM to complex with FT to promote the floral transition in Arabidopsis [18, 19]. FT, FD and 14‐3‐3 proteins are evolutionarily conserved in flowering plants [17, 21]. The crystal structures of the Arabidopsis and rice FACs have been determined, revealing the direct binding of FD to an ACGT‐bearing motif that is facilitated by FT [17, 20].

FT, first discovered in Arabidopsis, is found in all flowering plants examined so far [1, 22, 23, 24]. Multiple *FT* orthologs or homologs exist in various angiosperms, and the functions of *FT‐like* genes often have been diversified during recent evolution [25, 26]. In the long‐day plant sugar beet (*Beta vulgaris*), there are two *FT* paralogs including *BvFT1* and *BvFT2* with opposite function in the regulation of flowering time: *BvFT1* functions as a floral repressor and inhibits the expression of the potent floral inducer *BvFT2* [27]. In the facultative short‐day plant soybean (*Glycine max*), there are 12 *FT* homologs in six homoeologous pairs, among which four *FT‐like* genes including *GmFT1a*, *GmFT2a*, *GmFT4* and *GmFT5a* play important roles to regulate flowering [28]. Under short‐day (SD) conditions, both *GmFT2a* and *GmFT5a* are expressed at high levels to strongly promote the floral transition, whereas in LDs both genes are expressed at low levels, but are essential for eventual flowering after prolonged vegetative growth [29, 30]. Interestingly, both *GmFT1a* and *GmFT4* are highly expressed in LDs and function to inhibit the floral transition [31, 32]. *GmFT1a* overexpression results in the repression of both *GmFT2a* and *GmFT5a* and consequent late flowering in both SDs and LDs [31]. It seems that the functionally‐antagonistic *FT‐like* genes may regulate the expression of their opposing homologs to ensure the appropriate timing of flowering in response to environmental cues, but the underlying molecular mechanisms are unknown.

Plants fine‐tune the timing of their transition to flowering to optimize the balance between vegetative growth and reproductive success [2, 33]. A shorter vegetative growth period or early flowering typically results in inadequate accumulation of resources for maximal seed production [33]. On the other hand, a delay in flowering can result in missing the optimal timing and environmental conditions necessary for maximizing seed production in the field, as evidenced by overexpression of a floral inhibitor in rice resulting in a ten‐day delay in flowering and a significant reduction in grain yield in the paddy fields [34]. Because *FT* or *FT* relatives play a central role to promote the transition to flowering in angiosperms [1, 2], the proper level of FT production is crucial to balance vegetative growth and reproduction. Under inductive day lengths, the gradual accumulation of the CO protein toward the end of light period results in an increasing buildup of CO [5], which may cause an excessive transcriptional activation of *FT*, resulting in precocious transition to flowering under normal growth conditions. How day‐length induction of FT production is properly controlled to prevent its overproduction, is essentially unknown.

Here we report a negative feedback loop governing *FT* expression to prevent excessive FT production under inductive long days in Arabidopsis. We found that the bZIP transcription factor *FD* is expressed in leaf veins to downregulate *FT* expression and thus delay the floral transition. Upon the CO protein accumulation toward dusk, *FT* expression is strongly activated around dusk. We show that the FT protein complexed with FD (FD‐FT) recognizes several *cis*‐regulatory motifs in *FT* promoter to antagonize CO‐mediated *FT* activation. FD‐FT inhibits CO‐triggered *FT* promoter looping in transcriptional activation. Our results reveal that FT feedback downregulates its own expression to prevent its excessive induction by the photoperiod pathway, thereby precluding precocious transition to flowering in Arabidopsis. Furthermore, we found that the soybean *FT* homolog *GmFT4*, like *FT*, directly represses its own expression. Thus, the auto‐repression of *FT* or an *FT* homolog is a conserved mechanism to prevent excessive production of this potent floral regulator in flowering plants, ensuring the plants to flower at a proper time to balance vegetative growth with reproductive success under a given environmental setting. Moreover, we found that *GmFT4* directly represses the expression of *GmFT2a* and *GmFT5a* to inhibit soybean flowering, revealing a molecular mechanism for the cross‐regulation of antagonistic *FT‐like* genes in soybean and likely in other flowering plants.

## 2 Results

### 2.1 *FT* Represses Its Own Expression

**FIGURE 1:**
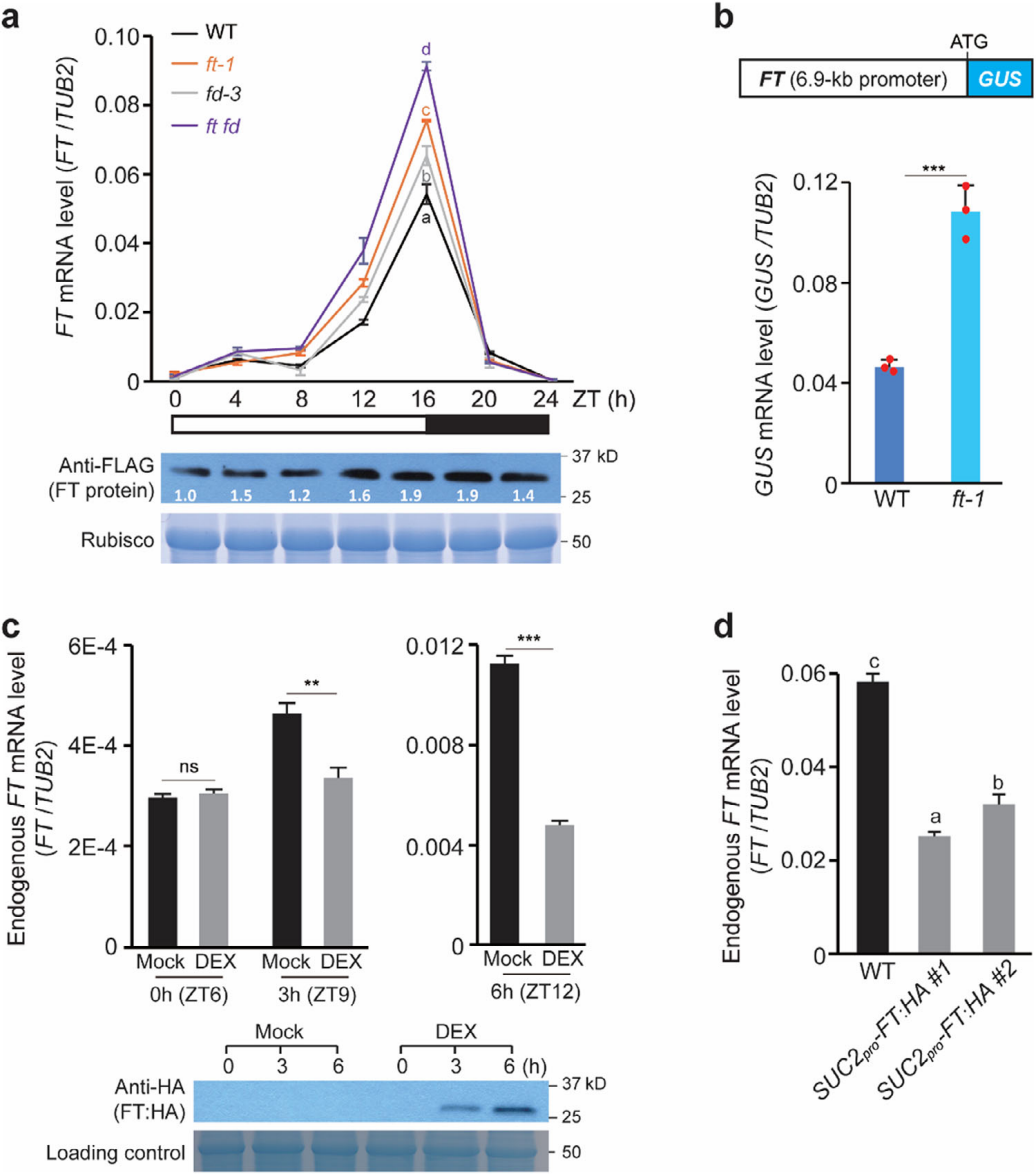
*FT* represses its own expression. a) *FT* expression in the rosette leaves of WT (Col‐0), *ft‐1*, *fd‐3* and *ft fd* over a 24‐h (h) LD cycle. Levels of *FT* or *ft* transcript were measured by quantitative PCR and normalized to the constitutively‐expressed *TUBULIN2* (*TUB2*), and values are means ± standard deviation (s.d.) of three biological replicates. Letters indicate statistically‐distinct means at ZT16 (one‐way ANOVA, *p* < 0.01). Shown on the panel bottom are the levels of FT:FLAG in rosette leaves over a LD cycle, examined by immunoblotting with anti‐FLAG (Rubisco serving as a loading control). Note that the first pair of rosette leaves from 14‐day (d)‐old seedlings were sampled. b) *FT_pro_‐GUS* expression in rosette leaves of the indicated T_1_ transgenic plants grown in LDs. Leaves (1^st^ pair) from 25 to 30 independent 14 d‐old T_1_ seedlings per line were harvested at ZT12 and pooled for RNA extraction in each sample. Levels of *GUS* transcript were normalized to *TUB2*, and values are means ± s.d. of three biological replicates (^***^ *p* < 0.001 from two‐tailed *t* test), and data points are overlaid on the bar graph. c) Levels of the endogenous *FT* transcript upon *FT:HA* induction by DEX in the seedlings grown in LDs. Seedlings were treated with DEX (20 µM) at ZT6, and subsequently were harvested at ZT9 and ZT12. Levels of *FT* transcript were normalized to *TUB2*, and values are means ± s.d. of three biological replicates (^**^ *p* < 0.01, ^***^ *p* < 0.001, and ns for not significant from two‐tailed *t* test). Levels of the FT:HA protein induced ectopically by DEX were examined by immunoblotting and shown in the panel bottom, with Rubisco as a loading control. d) Endogenous *FT* expression in rosette leaves of WT and *SUC2_pro_‐FT:HA* transgenic lines. Samples were harvested at ZT16. Values are means ± s.d. of three biological replicates. Letters in indicate statistically‐distinct means (one‐way ANOVA, *p* < 0.01).

It is well known that FT partners with the bZIP transcription factor FD to activate the expression of floral meristem identity genes to promote the floral transition [18, 19], and the FT‐FD complex has been thought to function mainly to activate target gene expression. In an effort to elucidate photoperiodic regulation of *FT* expression, we unexpectedly found that in a weak loss‐of‐function *ft* allele, *ft‐1* (in the Col background), bearing a missense *ft* mutation (glycine 171 to glutamate [22, 35]), the level of *ft‐1* transcript was higher than *FT* in the wild type Col‐0 (WT) over a long‐day cycle (Figure 1a). We further examined the stability of *ft‐1* and *FT* mRNAs, and found that the *ft‐1* transcript degraded moderately faster than the *FT* mRNA (Figure S1a). Thus, the increased level of *ft‐1* mRNA (relative to the native *FT*) is not attributed to its stability. These results together indicate that *FT* may repress its own expression.

The *ft‐1* in the Col background was generated from an introgression of the *ft‐1* allele in Landsberg *erecta* (L*er*) to Col through multiple backcrosses [35]. Given the presence of polymorphisms in the promoter regions between the *FT* locus in Col and the *ft‐1* from L*er* [36], we further examined whether *FT* represses its own expression, using a recombinant *FT* expression reporter gene in which the reporter gene *β‐GLUCURONIDASE* (*GUS*) is driven by a 6.9‐kb *FT* promoter (from Col‐0) [37]. *FT_pro_‐GUS* was introduced into WT and *ft‐1*; subsequently, we found that its expression was strongly increased in the leaves of *ft*, compared to WT (Figure 1b and Figure S1b,c). Thus, *FT* functions to repress its own transcription.

To further confirm that *FT* feedback represses its own expression, we constructed an *FT* induction line expressing a stringent glucocorticoid‐inducible *FT* expression cassette (*FT* tagged by the epitope *HA*, *FT:HA*, and driven by the chimeric promoter *pOp* [38]). Subsequently, *FT* expression was ectopically induced at a seedling stage by dexamethasone (DEX). We found that six hour (h) after DEX application, the endogenous *FT* expression was greatly repressed (Figure 1c). Thus, *FT* indeed represses its own expression.

*FT* is specifically expressed in the phloem cells in leaf veins [39]. We overexpressed *FT:HA* by the phloem‐specific promoter of *SUCROSE TRANSPORTER 2* (*SUC2*) [40]. *FT:HA* overexpression in leaf veins resulted in early flowering (Figure S1d), consistent with previous findings [16]. Next, we examined the endogenous *FT* expression in two independent transgenic lines of *SUC2_pro_‐FT:HA*, and found that the expression of native *FT* was greatly suppressed in these lines (Figure 1d). These results further confirm that *FT* represses its own expression in leaf veins. *FT* mRNA expression is rhythmically regulated under inductive LDs (16‐h night /8‐h night): strongly activated around dusk (ZT16), but repressed at night and into the early afternoon under controlled environment [3, 4]. We found that *FT* appears to repress its own expression from late afternoon until dusk (Figure 1a). Consistently, we found that the FT protein (FT:FLAG) accumulated in leaf veins at a relative high level from late afternoon until night (Figure 1a), in line with a previous study [41].

Taken together, our results suggest that upon the induction of *FT* expression by the LD pathway from late afternoon toward dusk, *FT* feedback represses its own expression.

### 2.2 FD is Expressed in Leaf Veins and Functions to Repress *FT* Expression

FT, a PEBP homolog unable to recognize DNA motifs by itself, typically complexes with FD or FD PARALOG (FDP) to regulate target gene expression [18, 19, 42]. Therefore, we measured *FT* mRNA levels in *fd* [18] and *fd ft* over a long‐day cycle, and found that *FT* expression is de‐repressed upon loss of *FD* function (Figure 1a), consistent with the notion that FT complexes with FD to repress its own expression. In addition, we observed that the level of *FT* de‐repression was moderately higher in *ft fd* than either single mutant (Figure 1a), which may be partly attributed to that *ft‐1* is a weak allele.

**FIGURE 2:**
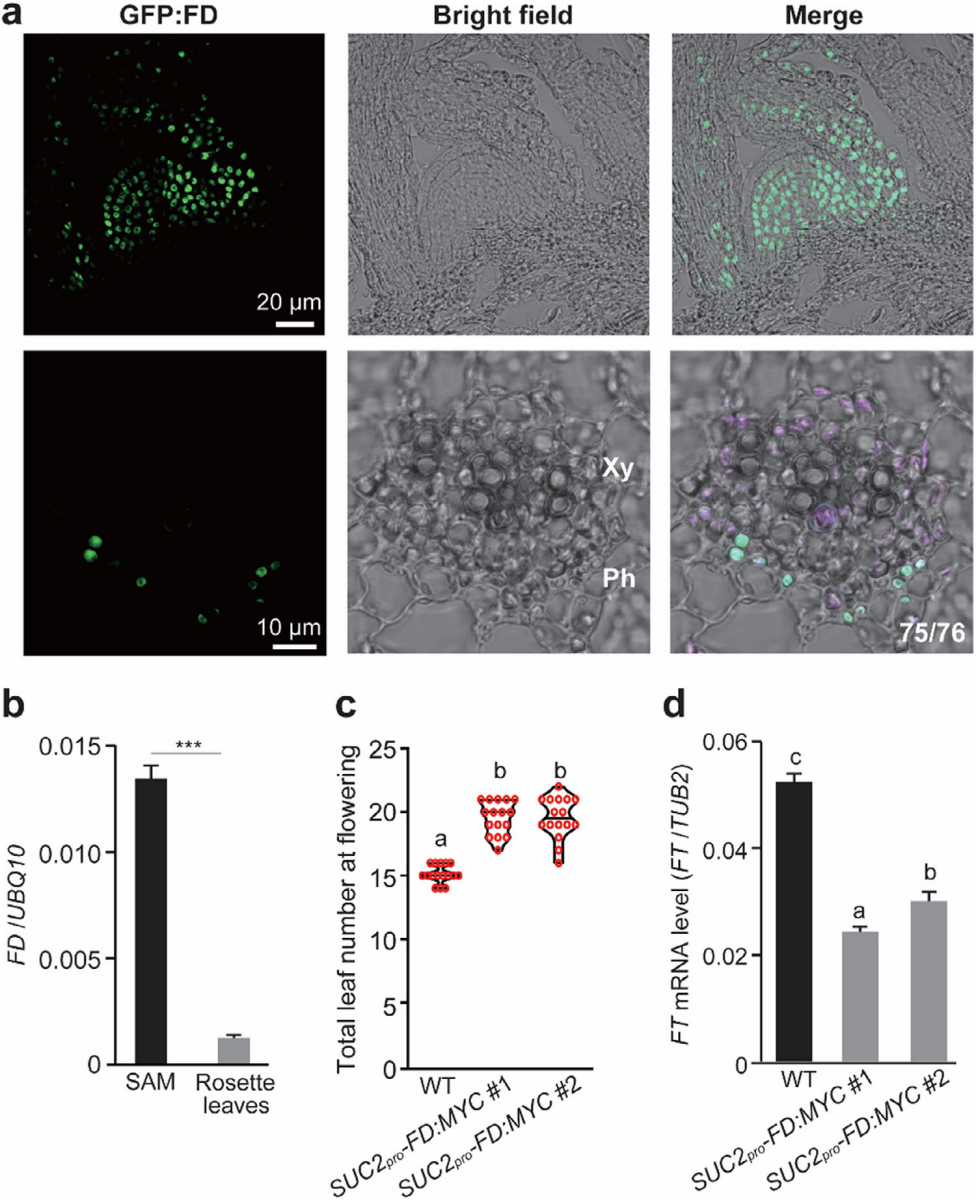
FD is expressed in leaf veins to repress *FT* expression. a) Confocal imaging of the GFP:FD localization in the shoot apical region (longitudinal sections in top panel) and major leaf veins (transverse sections in bottom panel). Ph, phloem; Xy, xylem; purple color for autofluorescence. Note that 75 out of the 76 examined leaf vein sections exhibit GFP fluorescence in the phloem tissue. b) Analysis of *FD* expression in the SAM region and rosette leaves. WT seedlings (grown in LDs) were sampled at ZT16. The levels of *FD* transcript were directly normalized to *UBQ10* (constitutively expressed in both SAM and leaves), and values are means ± s.d. of three biological replicates. c) Flowering times of *SUC2pro‐FD:MYC* lines (single‐locus and homozygous) grown in LDs. Total number of leaves formed prior to flowering was scored (16 plants per genotype). Data points are plotted on violin plots, with solid horizontal lines indicating medians. d) Analysis of *FT* expression in rosette leaves of WT and *SUC2pro‐FD:MYC* lines grown in LDs. 14‐d‐old seedlings were sampled at ZT16. Values are means ± s.d. of three biological replicates. Statistical significance of mean differences are assessed using two‐tailed *t* test in (b) (^***^ *p* < 0.001) and one‐way ANOVA in (c,d), with letters indicating statistically‐distinct means (*p* < 0.01).

FD is well known to function as a floral activator [18, 19]. It has been shown that *FD* is mainly expressed in the SAM where the FD protein functions as part of FAC to promote the floral transition [18, 19]. We reasoned that FD may be expressed in the phloem cells of leaf veins and partners with FT to repress *FT* expression in a feedback manner. First, we examined *FD* expression at a seedling stage, and found that it was expressed in both the SAM tissue and rosette leaves, though at a higher level in the SAM (Figure 2a,b). Next, to elucidate the role of FD in leaf veins, we examined a transgenic line of *FD_pro_‐GFP:FD* that can rescue the late‐flowering phenotype of the *fd* null mutant [43]. Using cryosectioning of leaf veins of GFP:FD, followed by confocal imaging, we observed that FD was localized in the SAM and phloem cells (companion cells) (Figure 2a and Figure S2), consistent with the notion that FT‐FD represses *FT* expression in leaf veins.

*FT* is specifically expressed in the phloem tissues of leaf veins. To further examine *FT* repression by FD in the phloem tissues, we introduced the transgene of *FD* (fused with the epitope *MYC*) driven by the phloem‐specific *SUC2* promoter (*SUC2_pro_‐FD:MYC*) into WT. In the first generation of transgenic plants, we found that over half of *FD:MYC* lines flowered later than WT (Figure S3a). Subsequently, we examined nine independent single‐locus transgenic lines (T_2_ generation) in which *FD:MYC* was overexpressed, and found that in seven lines *FT* expression in rosette leaves was repressed, resulting in late flowering (Figure S3b–d). We further examined two independent homozygous single‐locus *FD:MYC* lines (additional transgenic lines), and confirmed that *FT* expression was greatly repressed in these lines, resulting in late‐flowering (Figure 2c,d and Figure S3e). In addition, we observed that *CO* expression was not affected upon *FD:MYC* overexpression (Figure S3f). Thus, FD functions to repress *FT* expression in leaf veins to delay the transition to flowering.

Taken together, these results reveal that FD plays a dual role in the regulation of flowering time: downregulating *FT* expression in leaf veins, but complexing with the FT protein in the SAM to promote the floral transition.

### 2.3 FD‐FT Directly Represses *FT* Expression

**FIGURE 3:**
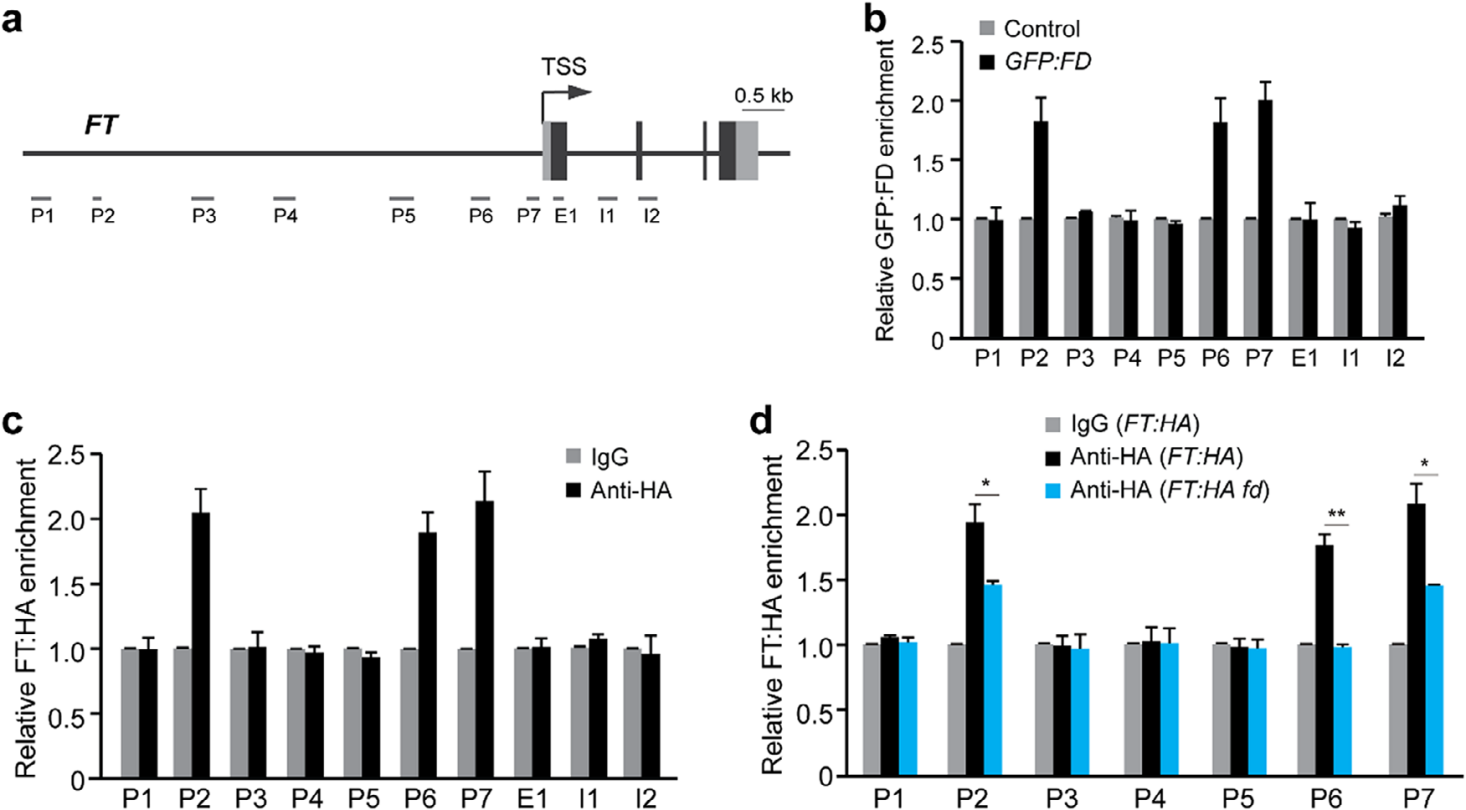
FD functions together with the FT protein to directly down‐regulate *FT* expression. a) Schematic diagram of the *FT* gene. Filled gray boxes, untranslated regions (UTRs); filled black boxes, coding regions; arrow, transcription start site (TSS). Chromatin immunoprecipitation (ChIP)‐examined regions are indicated with grey bars. b) ChIP analysis of GFP:FD enrichment at the *FT* locus. Immunoprecipitated *FT* DNA fragments by anti‐GFP were quantified by qPCR and normalized to the internal background gene *TUB2*. Shown are the fold enrichments of GFP:FD at each *FT* region in an *FD_pro_‐GFP:FD* line over background control (WT). c) ChIP analysis of FT binding to its own promoter. Total chromatin extracted from the *FT_pro_‐FT:HA* seedlings (in *ft‐1*) was immunoprecipitated using anti‐HA or anti‐IgG (background control). Relative FT fold enrichments at each examined *FT* region in the *FT:HA* line over the background control are shown. Notably, the *FT:HA* line carries a single‐locus transgene and the endogenous *ft‐1*, and anti‐IgG serves as a background control for ChIP. d) FD is required for FT binding to its own promoter regions, as revealed by ChIP‐qPCR with anti‐HA. Relative FT:HA fold enrichments in each *FT* promoter regions over the background control anti‐IgG are presented. b‐d) Samples were harvested at ZT16. Values are means ± s.d. of three biological replicates. Two‐tailed *t* test was conducted in (d), with significance levels indicated as ^*^ *p* < 0.05 and ^**^ *p* < 0.01.

To explore how *FT* regulates its own expression, we generated a transgenic line expressing an FT protein (tagged with HA) driven by *FT* promoter (*FT_pro_‐FT:HA*), which partly rescued the late flowering phenotype of *ft‐1* (Figure S1e). Next, we conducted chromatin immunoprecipitation (ChIP) assays, and found that FT:HA was strongly enriched in a distal region and two proximal regions of *FT* promoter (Figure 3a,c). Thus, FT directly regulates its own expression.

FT, lacking a DNA‐binding domain, complexes with FD to regulate target gene expression. In a previous study of genome‐wide occupancy of FD, it was found that FD occupies *FT* promoter [44]. We further conducted ChIP assays using the transgenic seedlings expressing the functional *FD_pro_‐GFP:FD*, and found that FD specifically bound the *FT* promoter regions where the FT protein binds (Figure 3b). This is consistent with that FD complexes with FT to repress *FT* expression in leaf veins.

Next, we determined whether the FD protein is required for FT binding to its own promoter regions. We first introduced *fd* into an *FT:HA* line by genetic crossing to generate an *FT:HA fd* line. Subsequent ChIP assays revealed that the FT enrichments at both distal and proximal *FT* promoter regions were strongly reduced upon loss of *FD* function (Figure 3d). Thus, FD indeed is required for FT binding to its own promoter. It has been shown that in the FAC complex FD directly binds DNA, whereas FT stabilizes the complex and also binds DNA [17, 20]. We further explored that whether FT may be partly required for FD binding *FT* promoter regions by conducting ChIP with an *GFP:FD ft* line, and found that loss of *FT* function resulted in an apparent reduction of FD enrichment in *FT* promoter regions (Figure S3g). These results, together with that both FT and FD bind the same *FT* promoter regions, reveal that FT complexes with FD to directly regulate its own expression.

### 2.4 FD Recognizes Three DNA motifs in *FT* Promoter to Repress Its Expression

**FIGURE 4:**
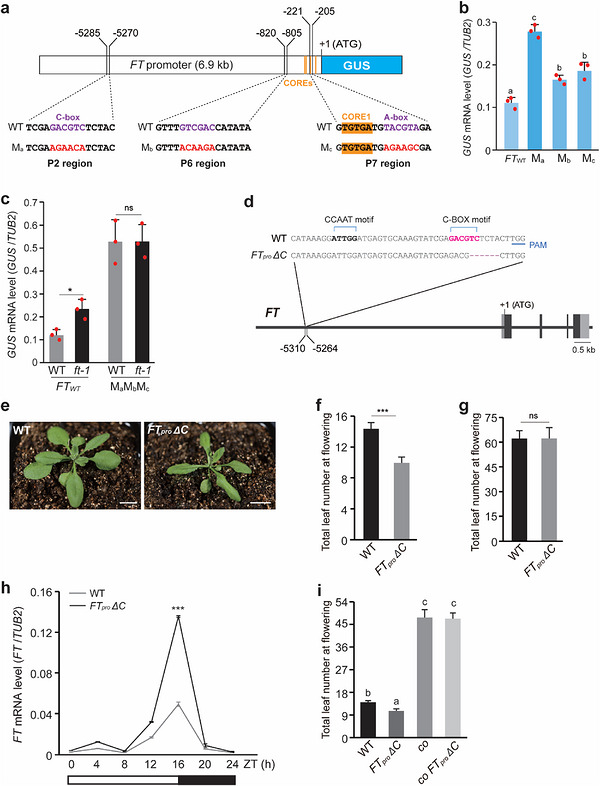
Characterization of the *cis*‐regulatory DNA motifs for *FT* auto‐repression. a) Schematic representation of the recombinant *FT_pro_‐GUS* reporter genes consisting of the GUS coding region driven by a 6.9‐kb *FT* promoter or a mutated *FT* promoter. M_a_, M_b_ and M_c_ denote mutated FD‐binding motifs in *FT* promoter. A of ATG as +1. b and c) Analysis of *GUS* expression in rosette leaves of *M_a_*, *M_b_*, *M_c_* (b) and *M_a_M_b_M_c_* (*mFT_pro_‐GUS*) seedlings (c) grown in LDs. *M_a_*, *M_b_* and *M_c_* are in the WT background. *M_a_M_b_M_c_* was introduced into both WT and *ft‐1*. The first pair of rosette leaves from 25 to 30 independent T_1_ seedlings (14‐d‐old) per construct were harvested at ZT16, and pooled for RNA extraction in each sample. Levels of *GUS* transcript were quantified by RT‐qPCR and normalized to *TUB2*. Values are means ± s.d. of three biological replicates, and data points are overlaid on bar graphs. d) Schematic illustration of CRISPR/Cas9‐mediated mutagenesis of the C‐box motif in the distal *FT* promoter. *FT_pro_ ΔC* bears a 6‐bp deletion, including 2 bp from the C‐box. PAM, protospacer adjacent motif. Note that NF‐Y recognizes the CCAAT motif to activate *FT* expression. e) Early‐flowering phenotype of *FT_pro_ ΔC* in LDs. Scale bars, 1 cm. f and g) Flowering times of WT and *FT_pro_ ΔC* in LDs (f) and SDs (g). 16–20 plants per genotype were scored, and bars for s.d. h) Diurnal expression of *FT* mRNA in rosette leaves of WT and *FT_pro_ ΔC* over a 24‐h LD cycle. Levels of *GUS* transcript were directly normalized to *TUB2*. Values are means ± s.d. of three biological replicates. i) Flowering times of the indicated lines grown in LDs. Total number of leaves from around 12 plants per line was scored, and bars indicate s.d. b‐c,f‐i) Two‐tailed *t* tests were conducted in (c,f‐h), *p < 0.05, ***p < 0.001, and ns for not significant; in addition, one‐way ANOVA was conducted in (b,i), with letters indicating statistically‐significant differences (*p*< 0.01).

The bZIP transcription factor FD recognizes ACGT or TCGA‐containing motifs in target promoters to regulate gene expression [18, 19, 43]. We further analyzed the *FT* promoter regions bound by FT‐FD, and identified at least one putative FD‐binding motif in each region, including C‐box (5’‐GACGTC‐3’), GTCGAC motif and A‐box (5’‐TACGTA‐3’) (Figure 4a) [17, 18, 43]. Next, we mutagenized these motifs in the *FT_pro_‐GUS* reporter gene: *M_a_* with mutated distal C‐box, *M_b_* with mutated GTCGAC motif, and *M_c_* with mutated A‐box (Figure 4a). The mutation of each motif resulted in varying degrees of *FT_pro_‐GUS* de‐repression with the mutation of the C‐box motif located in the distal *FT* promoter region exhibiting the greatest effect (Figure 4b). Furthermore, the mutant *FT_pro_‐GUS* (*mFT_pro_‐GUS*) in which all three putative FD‐bound motifs were mutated exhibited a great de‐repression (compared to wild‐type *FT_pro_‐GUS* in the WT background: over four‐fold increase) (Figure 4c). Taken together, these results show that all three FD‐bound motifs are partly required for *FT* repression with the C‐box motif in the distal *FT* promoter region playing a major role.

To confirm that FD indeed binds to the identified motifs in *FT* promoter, we first constructed *GFP:FD FT_WT_‐GUS*, *GFP:FD M_b_*, and *GFP:FD M_c_* by genetic crossing. Subsequent ChIP experiments using the F_1_ seedlings, revealed that the enrichments of FD in both the mutated GTCGAC motif‐containing region and mutated A‐box region were reduced (Figure S4a–c). Thus, FD indeed binds these two motifs in *FT* promoter. In addition, we also confirmed that FD binds to the distal C‐box in *FT* promoter (described in next section).

Next, to determine whether the great de‐repression of *mFT_pro_‐GUS* is attributed to a disruption of *FT* auto‐repression by FD‐FT, we introduced this transgene into *ft‐1*. Subsequent measurements of *GUS* expression revealed that the level of *mFT_pro_‐GUS* de‐repression in both *ft* and WT was nearly identical (Figure 4c), suggesting that these *cis*‐regulatory motifs mediate *FT* auto‐repression. Interestingly, we noticed that the extent of *mFT_pro_‐GUS* de‐repression in *ft‐1* was greater than the extent of *FT_pro_‐GUS* de‐repression in *ft* (Figure 4c). This may be attributed to that the ft‐1 protein is still partially functional.

The major motif C‐box for *FT* repression is located 18 bp downstream of the essential enhancer element (CCAAT) for CO‐mediated *FT* activation [11, 12]; hence, it was of great interest to explore the role of this distal C‐box in the *FT* repression by FT‐FD. Using a CRISPR/Cas9‐based method, we generated a homozygous mutant, *FT_pro_ ΔC*, with a six‐bp deletion (5’‐TCTCTA‐3’) that removed two bp of the C‐box (5’‐GACGTC‐3’) (Figure 4d). Next, we measured *FT* expression in this mutant over a LD cycle, and found that it was strongly de‐repressed in *FT_pro_ ΔC* compared to WT at ZT16 (Figure 4h). This confirms that the C‐box indeed plays an important role for *FT* repression. The *FT_pro_ ΔC* mutant exhibited early flowering in LDs, but not in SDs (Figure 4e–g). This long day‐dependent early flowering phenotype is consistent with the role of C‐box in *FT* auto‐repression, namely, the long day‐induced CO accumulation is required for *FT* repression. Note that the CO protein is degraded rapidly in SDs [5]. We further introduced a *co* null mutation into *FT_pro_ ΔC*, and found that *co FT_pro_ ΔC* exhibited the same late‐flowering phenotype as *co* (Figure 4i), confirming that the early‐flowering of *FT_pro_ ΔC* depends on the *CO* function in LDs. Taken together, these results show that the C‐box plays an important role for long day‐dependent *FT* auto‐repression.

We further assessed the biological function of the C box‐mediated *FT* auto‐repression by measuring the indicators of overall plant growth and development, including biomass and seed yield [45]. We found that the early flowering in the *FT_pro_ΔC* line leads to a significant reduction by about 50% in biomass as well as a decreased seed yield compared to WT in LDs (Figure S5a–c). Thus, the C box‐mediated *FT* auto‐repression generates a proper level of FT production under inductive long days, to balance vegetative growth with reproduction and thus maximize plant production.

### 2.5 FD Binds the C‐Box in the Distal *FT* Promoter to Inhibit Promoter Looping

**FIGURE 5:**
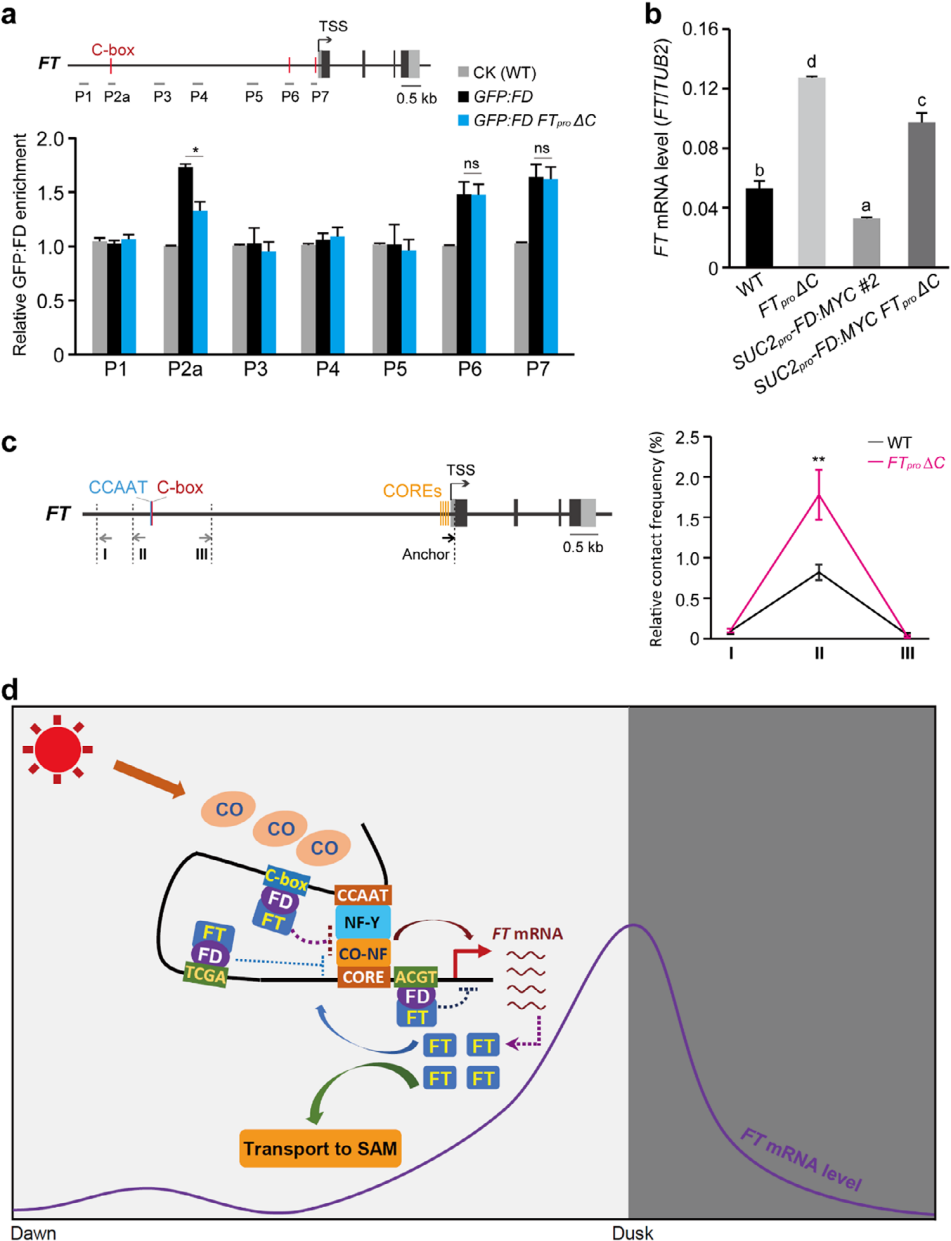
FT‐FD binds the C‐box in distal *FT* promoter to disrupt *FT* promoter looping and attenuate CO‐NF‐mediated *FT* activation under inductive LDs. a) ChIP analysis GFP:FD binding to *FT* promoter regions upon C‐box deletion (ΔC). Shown are relative fold enrichments of GFP:FD across *FT* promoter over a background control (WT). Samples were harvested at ZT16. Values are means ± s.d. of three biological replicates. Two‐tailed *t* tests were conducted to evaluate means differences (* *p* < 0.05 and ns for not significant). b) *FT* expression in rosette leaves of indicated lines. The levels of *FT* transcript were quantified by RT‐qPCR and normalized to *TUB2*. Values are means ± s.d. of three biological replicates. Letters mark statistically significant differences (one‐way ANOVA, *p* < 0.01). c) Quantitative 3C analysis of *FT* promoter looping between the distal CCAAT enhancer and the proximal CORE region in WT and *FT_pro_ ΔC* seedlings grown in LDs. On the left is a schematic diagram of *FT* gene, with vertical dotted lines to denote *Dpn II* cutting sites; the CORE region is set as the anchor, and arrows next to the cutting sites indicate primers. Relative contact frequencies between the anchor and indicated regions were calculated as described in the Methods. Samples were harvested at ZT16. Values are means ± s.d. of three biological replicates, and evaluated by two‐tailed *t* test (^**^ *p* < 0.01). d) A working model for auto‐downregulation of the florigen FT production in Arabidopsis under inductive LDs. From late afternoon to dusk, increasing buildup of the CO protein leads to the increased assembly of the transcriptional activator complex CO‐NF; in addition, the distal CCAAT enhancer recognized by NF‐Y frequently interacts with the proximal CORE region bound by CO‐NF, resulting in promoter looping for transcriptional activation. The FT proteins synthesized in leaf veins (companion cells) complex with FD, and the FD‐FT complex binding the distal C‐box (next to the CCAAT enhancer), downregulates the looping frequency of *FT* promoter; in addition, FD‐FTs binding the A‐box within the CORE region and the GTCGAC motif close to CORE also act to attenuate CO‐NF‐mediated *FT* activation. In short, there is a ‘braking' mechanism ensuring a proper level of FT production to balance vegetative growth with reproduction for improved biomass and seed yield under inductive photoperiods.

To understand how the regulatory module of FT‐FD‐C box represses *FT* expression, we first determined whether FD binds the C‐box in distal *FT* promoter by ChIP using the GFP:FD and GFP:FD *FT_pro_ ΔC* lines. The enrichment of FD at *FT* promoter was reduced specifically in the C box‐containing region upon the partial deletion of C‐box, confirming that FD indeed binds this C‐box (Figure 5a). Furthermore, we determined whether C‐box is crucial for *FT* repression by FD by introducing *FT_pro_ ΔC* into a late‐flowering *SUC2_pro_‐FD:MYC* line. We found that *SUC2_pro_‐FD:MYC* was not able to suppress the *FT_pro_ ΔC*‐mediated *FT* de‐repression, and that the *SUC2_pro_‐FD:MYC FT_pro_ ΔC* line flowered earlier like *FT_pro_ ΔC* (Figure 5b and Figure S5d). In addition, we found that the transcriptional repression of endogenous *FT* by *SUC2_pro_‐FT* was relieved by the C‐box mutation in *FT_pro_ ΔC* (Figure S5e). These results together show that FT‐FD binds the C‐box in distal *FT* promoter to mediate *FT* auto‐repression.

*FT* promoter looping between the distal CCAAT‐bearing enhancer region and the proximal CO‐bound region with CO‐responsive elements (COREs) is essential for CO activation of *FT* expression in inductive LDs [11, 12]. The FT‐FD‐bound C‐box is next to CCAAT. We speculated that the FT‐FD‐C‐box regulatory module may interfere with CO‐triggered *FT* promoter looping to downregulate its expression. Hence, we measured the interaction frequencies between the proximal CORE region and distal regions of the *FT* promoter in WT and *FT_pro_ ΔC*, using the chromosome conformation capture (3C) approach. We found that the looping frequency between the CCAAT enhancer region and the CORE region was greatly increased upon the C‐box removal (Figure 5c). Thus, the FT‐FD‐C‐box regulatory module indeed inhibits the interaction of the distal CCAAT enhancer with the proximal promoter region to downregulate CO‐mediated *FT* activation in inductive LDs.

Polycomb‐repressive complex 2 (PRC2) plays an important role in *FT* repression [46]. We found that the FT protein interacted with the PRC2 subunit CURLY LEAF (CLF) in yeast cells (Figure S6a). Subsequent ChIP experiments show that CLF enrichment on *FT* promoter chromatin was slightly reduced (by approximately 20%) in several examined regions at dusk in the *ft fd* mutant (Figure S6b). These results indicate a limited role of PRC2 in *FT* auto‐repression.

Our results thus far show that FD‐FT binds *FT* promoter, particularly the distal C‐box, to antagonize CO‐mediated *FT* activation and thus feedback downregulate *FT* expression (Figure 5d). This prevents an excessive *FT* induction by the photoperiod pathway in Arabidopsis.

### 2.6 An *FT*‐Like Gene Represses Its Own Expression and Other *FT* Homologs in Soybean

**FIGURE 6:**
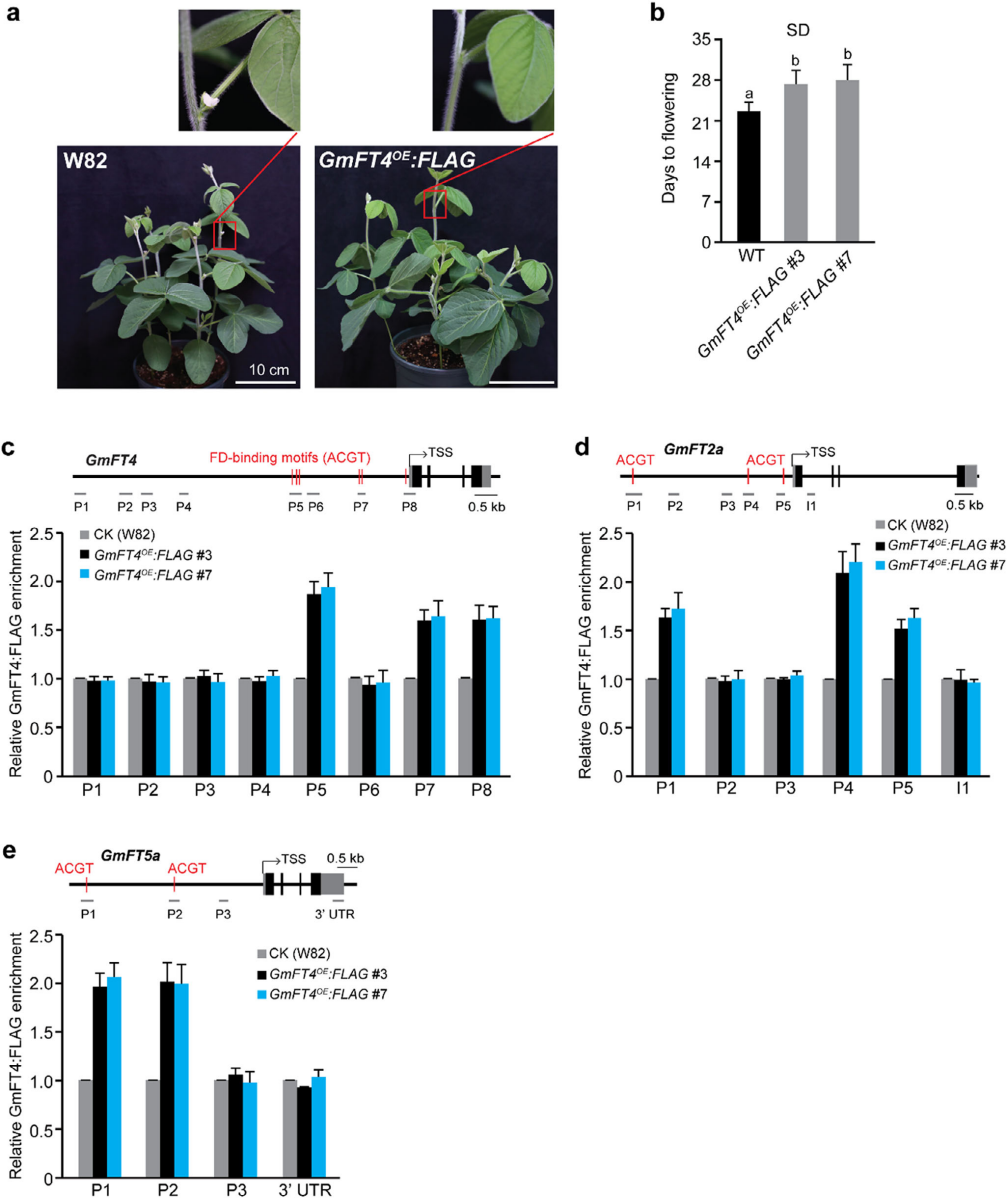
G*mFT4* directly represses its own expression as well as *FT‐like* genes in soybean. a) Phenotypes of the transgenic lines overexpressing *GmFT4:FLAG* (*GmFT4^OE^:FLAG*) grown for 21 days after cotyledon emergence (DAE) in SDs. Scale bars, 10 cm. b) Flowering times of *GmFT4^OE^:FLAG* lines grown in SDs. About 17 plants for each line were scored. Kruskal‐Wallis nonparametric analysis with Dunn's multiple comparison tests was conducted, and letters indicate statistically significant differences (*p* < 0.01). c‐e) ChIP‐qPCR analysis of GmFT4 enrichment at the *GmFT4* (c), *GmFT2a* (d) and *GmFT5a* loci (e). On top of each panel is a schematic drawing of a soybean *FT‐like* gene (filled gray boxes for UTRs, and filled black boxes for coding regions), with red lines to denote putative FD‐binding motifs and grey bars for ChIP‐examined regions. Total chromatin was extracted from the leaves of two independent *GmFT4^OE^:FLAG* lines grown in SDs (harvested at ZT8), followed by immunoprecipitation with anti‐FLAG. DNA fragments were quantified by qPCR and normalized to the internal control *Glycine max ACTIN 11* (*GmACT11*). Shown are relative fold enrichments of GmFT4:FLAG over background control (the non‐transgenic background *Williams 82*). Values are means ± s.d. of three biological replicates.

To elucidate whether *FT* auto‐repression is a conserved mechanism to regulate *FT‐like* gene expression in flowering plants, we explored the regulation of *FT‐like* genes in soybean, a facultative short‐day plant with multiple *FT‐like* genes [28]. In response to SDs, both *GmFT2a* and *GmFT5a* are expressed at relative high levels to promote flowering, whereas under LDs, *GmFT1a* and *GmFT4* are highly expressed to inhibit the floral transition, resulting in late flowering [28]. Recently, it has been shown that the relative expression levels between *GmFT4* and *GmFT2a*/*GmFT5a* play an important role to determine soybean flowering under different day lengths [47]. We generated several transgenic lines in which *GmFT4* (tagged with *FLAG*) was overexpressed (*GmFT4^OE^:FLAG*) (Figure 6a and Figure S7a). We found that in both SDs and LDs, *GmFT4* overexpression strongly repressed the expression of the endogenous *GmFT4* (Figure S7b,d). Next, we conducted ChIP assays with *GmFT4^OE^:FLAG* seedlings, and found that GmFT4 was enriched at three *GmFT4* promoter regions bearing putative FD‐binding motifs (ACGT‐containing elements) (Figure 6c). Note that FD is evolutionarily conserved in soybean [28]. Thus, like FT‐FD in Arabidopsis, GmFT4 directly represses its own expression in soybean. This reveals that the auto‐repression of *FT* or an *FT* homolog is a conserved mechanism to prevent its excessive production in both Arabidopsis and soybean.

Functionally antagonistic *FT‐like* genes have been widely found in flowering plants, the floral repressor *GmFT4* may repress the expression of *GmFT2a* and *GmFT5a* to inhibit flowering in LDs [28, 47]. Indeed, we found that *GmFT4* overexpression represses both *GmFT2a* and *GmFT5a* expression, but not *GmFT1a* expression (Figure S7c,e–g), resulting in a delay in flowering under SDs (Figure 6b). Furthermore, we uncovered that GmFT4:FLAG was enriched at multiple *GmFT2a* and *GmFT5a* promoter regions bearing FD‐binding motifs (with the ACGT core) (Figure 6d,e), revealing a direct regulation by GmFT4. These results are consistent with the notion that under LDs the highly‐expressed GmFT4 directly represses the expression of both *GmFT2a* and *GmFT5a* to inhibit flowering in soybean. Thus, in a flowering plant with multiple *FT* paralogs or homologs, an FT‐like protein may not only downregulate its own expression, but also directly modulate the expression of its homologs to determine an appropriate timing to flower in response to endogenous and/or environmental signals such as day‐length changes.

## 3 Discussion

In this study, we have found that a negative feedback loop regulates the expression of the florigen *FT* to prevent excessive FT production and precocious flowering under inductive photoperiods. We show that the bZIP transcription factor FD is expressed in leaf veins and that FT‐FD specifically binds several ACGT/TCGA‐containing motifs in *FT* promoter to downregulate or attenuate CO‐mediated *FT* activation from late afternoon to dusk when the CO protein is built up with increasing light period in inductive LDs. We further show that FT‐FD functions to inhibit CO‐triggered *FT* promoter looping in transcriptional activation. Thus, FT‐FD antagonizes the transcriptional *FT* activation by CO‐NF, leading to a downregulation of *FT* expression. Together, these findings define an auto‐repression mechanism to prevent excessive FT production upon the accumulation of the photoperiod pathway output (CO), conferring an appropriate timing to flower in response to an inductive long‐day signal. Furthermore, we have found that in the facultative short‐day plant soybean, the *FT* homolog *GmFT4*, like *FT* in Arabidopsis, directly represses its own expression. Thus, the auto‐repression of *FT* or an *FT* homolog is a conserved mechanism to prevent excessive production of this potent floral regulator in flowering plants.

The coincidence of the circadian‐regulated *CO* transcription and exposure to light (blue and far‐red light) gives rise to an increasing buildup of the CO protein toward the end of daytime under inductive long‐day conditions [1, 2]. The fluctuating light conditions in a given growth period, including varying light quality, intensity, and duration, such as extended or reduced daylight exposure, are expected to lead to an over‐ or under‐accumulation of the CO protein, which may cause excessive or insufficient transcriptional activation of *FT* expression. The auto‐repression of *FT* provides an important layer of regulation to buffer the fluctuations in CO‐mediated *FT* activation. This ensures that the florigen FT is produced at an appropriate level to balance vegetative growth with reproduction under a particular season, as evidenced by that a disruption of auto‐repression of *FT* results in a great reduction in biomass and seed yield under a controlled environmental setting. The FT proteins are translocated from leaves to the SAM, and the movement is mediated by FT‐INTERACTING PROTEIN 1 (FTIP1), SODIUM POTASSIUM ROOT DEFECTIVE 1, and several additional proteins [16, 48, 49]. Interestingly, loss of *FTIP1* function leads to a reduction in *FT* mRNA expression, suggesting that the disruption of FT movement may feedback repress *FT* expression [16]. This is consistent with the role of *FT* in its own repression.

*FT*, encoding a PEBP protein, is evolutionarily conserved in flowering plants [24, 26]. The *PEBP* gene family in angiosperms consists of three clades including *MOTHER OF FT AND TFL1* (*MFT*)‐*like*, *TERMINAL FLOWER 1* (*TFL1*)*‐like* and *FT‐like*. *TFL1‐like* genes typically function as floral repressors, whereas *FT‐like* genes often act as floral inducers [25, 26]. During recent evolution of *FT‐like* genes through gene duplication and evolutionary innovation in flowering plants, the *FT‐like* clade has generated antagonistic regulators that function to fine‐tune the timing of floral induction and other developmental transitions, in response to environmental signals [25, 26]. For instance, the two *FT* paralogs in sugar beet function oppositely to regulate flowering with *BvFT1* repressing *BvFT2* expression [27]. In the biennial crop onion (*Allium cepa L*.), there are four *FT* paralogs, both *AcFT2* and *AcFT1* promote the floral transition and bulb formation in LDs, respectively, whereas *AcFT1* expression is repressed by *AcFT4* to prevent bulb formation in SDs [50]. In the SD plant potato (*Solanum tuberosum*), the *FT‐like* gene *SELF PRUNING 5G* (*StSP5G*) represses the expression of the *FT* ortholog *StSP6A* in LDs to prevent tuberization [51]. In soybean, long‐day photoperiods lead to a high‐level expression of *GmFT1a* and *GmFT4*, which repress the expression of the floral activators *GmFT2a* and *GmFT5a*, resulting in an inhibition of the floral transition [28]. Furthermore, it has been shown that upon loss of *GmFT2a* or *GmFT5a* function, *GmFT5a* or *GmFT2a* expression in SDs is upregulated, respectively, leading to genetic compensation [52]. This suggests that *GmFT2a* and *GmFT5a* reciprocally repress each other's expression. Taken together, it is common in flowering plants that an *FT‐like* gene represses the expression of its homologs to fine‐tune developmental transitions. In this study, we have found that *GmFT4* directly represses the expression of *GmFT2a* and *GmFT5a* to inhibit soybean flowering, pointing a molecular mechanism for the cross‐regulation of antagonistic *FT‐like* genes in soybean and likely other flowering plants.

It has been long known that FD is expressed preferentially in the SAM and functions as a potent inducer of the floral transition [18, 19]. Intriguingly, the constitutive expression of *FD* by the *35S* promoter has yielded conflicting flowering phenotypes (early vs. late flowering) in Arabidopsis [18, 19], likely due to a variation of *FD* expression. In this study, we have uncovered that FD is expressed in leaf veins and complexes with the FT protein to repress *FT* expression and thus delay the floral transition under inductive LDs. We show that *SUC2_pro_‐FD:MYC* gives rise to a delay in flowering in a majority of transgenic lines (Figure S3a–d). For example, in seven out of the nine single‐locus *SUC2_pro_‐FD:MYC* lines, *FT* expression is downregulated in leaf veins, while in the remaining two lines with a high level of *FD* expression, *FT* is expressed at a level similar to WT. Not surprisingly, a previous study reports that a *SUC2_pro_‐GFP:FD* line exhibits no discernible flowering phenotype (compared to the Col‐0 background) [43].

The variation of flowering times among various transgenic lines expressing *SUC2_pro_‐FD* highlights the delicate and complex roles of *FD* in flowering‐time regulation. A recent study shows that FD alone tends to condensate and that the interaction of FD with 14‐3‐3 reduces its condensation in the SAM cells [20]. It is likely that FD‐FT may complex with 14‐3‐3 in leaf veins to repress *FT* expression, an interesting topic worthy of future pursuit. It is intriguing that in two *SUC2_pro_‐FD:MYC* lines with the highest *FD* expression levels, *FT* expression is not downregulated (Figure S3b,c). Given the propensity of FD for condensation, a high level of the FD protein may drive concentration‐dependent phase separation that disrupts FD function; in addition, FD may sequester a limiting partner of the FD‐FT‐containing complex to prevent its function in *FT* repression. Noteworthily, in most *SUC2_pro_‐FD:MYC* lines, the elevated levels of FD in leaf veins are predicted to retain more FT proteins in the nucleus to repress *FT* expression, through inhibiting the *FT* promoter looping and likely additional mechanisms.

We have uncovered that FD plays a dual role in long‐day induction of flowering: highly expressed in the SAM and complexed with FT to activate floral initiation, and moderately expressed in leaf veins and complexed with FT to prevent FT overproduction in inductive photoperiods, ensuring an appropriate photoperiodic flowering response. Of note, owing to technical limitations, the FD‐FT interaction in phloem cells was not directly verified in this study. Certainly, the dynamics of FT‐FD (and possibly FT‐FD‐14‐3‐3) interaction in leaf veins over a LD cycle will be an interesting topic for future study. In addition to FD, FD homologs may be involved in *FT* autorepression. We found that the FT enrichment at two of the three FD‐bound regions in the *FT* promoter is partly dependent on FD (Figure 3d), suggesting that FD homologs such as FDP and other bZIP transcription factors, may be involved in recruiting FT to target regions, as evidenced by that FDP partners with FT to promote the floral transition in SAM [42].

FD is evolutionarily conserved in flowering plants [21, 28]. In soybean, FD‐like proteins complex with FT‐like proteins to regulate the expression of flowering‐regulatory genes [28]. We have found that GmFT4 is enriched specifically in the regions bearing FD‐binding motifs (with the ACGT core) in *GmFT4* promoter, suggesting that GmFT4 complexes with a FD‐like protein to repress its own expression. These findings collectively reveal that the FT‐FD complex can function as a transcriptional repressor to form an autoregulatory loop that mediates the feedback repression of *FT* or *FT‐like* genes in flowering plants. Noteworthily, a FAC, formed in rice leaves, can feedback inhibit *Hd3a* expression indirectly [53], suggesting that multiple regulatory loops are involved in transcriptional regulation of *FT* or *FT‐like* genes to fine‐tune flowering time, in response to environmental cues.

In summary, we have uncovered an auto‐repression mechanism to prevent excessive production of the florigen FT in Arabidopsis and likely in other flowering plants under inductive photoperiods. This results in an appropriate level of the mobile FT or FT‐like proteins generated in leaf veins, to optimize vegetative growth for improved seed yield or reproductive success by preventing precocious transition to flowering in response to photoperiodic induction.

## 4 Materials and Methods

### 4.1 Plant Materials and Growth Condition

All Arabidopsis mutants and transgenic lines used in this study are in the Columbia (Col‐0) background. *ft‐1* (in the Col background), *fd‐3*, *co‐9* and the transgenic line *FD_pro_‐GFP:FD* have been described previously [18, 35, 43, 54]. Seeds were germinated on agar plates with half‐strength MS media, and plants were grown at around 22°C in LDs (16‐h light/8‐h dark) or SDs (8‐h light/16‐h dark) with cool white light.

The soybean cultivar *Williams 82* was used in this study. Plants were grown at around 26°C under LDs (16‐h light /8‐h dark) or SDs (12‐h light/12‐h dark).

### 4.2 Plasmid Construction

The previously described *FT_pro_*‐*GUS* fragment [37] was cloned into the binary vector *pBGW* [55] via Gateway technology (Invitrogen). To generate *FT_pro_‐FT:FLAG* and *FT_pro_‐FT:HA*, a 11.0‐kb genomic fragment of *FT* (8.9 kb upstream of ATG plus the 2.1‐kb genomic coding sequence except for the stop codon) was fused in frame with three copies of FLAG or one copy of HA at 3’ end, respectively, and the fusions were cloned into *pHGW* [55] using Gateway technology. To create *SUC2_pro_‐FT:HA* and *SUC2_pro_‐FD:MYC*, the 2.0‐kb *AtSUC2* promoter was first fused with 0.5‐kb *FT* or 0.8‐kb *FD* coding sequence (without the stop codon) tagged with three copies of *HA* or four copies of *MYC* (at 3’ end), respectively; subsequently, the fusions were cloned into the binary vector *pBGW*.

To construct the sgRNA*^FT^*‐CRISPR/Cas9 expression cassette, an sgRNA fragment targeting an *FT* promoter region was introduced into *psgR‐Cas9‐At* at *Bbs I* [56], and then the *sgRNA*‐*Cas9* cassette was cloned into the binary vector *pCAMBIA1300* at *Hind III* and *EcoR I*. For *35S_pro_‐GmFT4:FLAG* construction, the 0.5‐kb coding sequence of *GmFT4* (*Glyma.08G363100*) from the reference cultivar *Williams 82* was fused in‐frame with three copies of FLAG at 3' end; subsequently, the fusion was cloned into the binary vector *pB2GW7* [55] using Gateway technology.

To generate the dexamethasone (DEX)‐inducible *p35S‐GR:LhGR4‐pOp‐FT:HA* construct, the chimeric promoter *pOp* in the *pOp/LhG4* system [38] was first fused with the 0.5‐kb *FT* coding sequence (without the stop codon) tagged with three copies of HA at 3' end; subsequently, the *pOp‐FT:HA* cassette located downstream of *p35S‐GR:LhGR4* was cloned into the binary vector *pHGW* [55].

### 4.3 Gene Expression Analysis

Total RNAs were extracted from leaves or seedlings grown in LDs, with *Eastep Super Total RNA* extraction kit (Promega) according to the manufacturer's instructions. DNA digestion and reverse transcription were conducted using *HiScript III 1st Strand cDNA Synthesis kit* (+gDNA wiper) according to the manufacturer's instructions (Vazyme); subsequently, real‐time quantitative PCR (qPCR) was performed on an *ABI QuantStudio5 Flex* real‐time PCR system using a SYBR qPCR master mix (Vazyme). Transcript levels of gene of interest were normalized to a constitutively‐expressed reference gene in Arabidopsis or soybean. Primers are listed in Table S1. Notably, the endogenous levels of *FT* transcript are quantified using a pair of primers to amplify a 3’‐UTR region.

### 4.4 Yeast Two‐Hybrid Assay

Yeast two‐hybrid assays were conducted according to the instructions from *Matchmaker GAL4 Two‐Hybrid System* (Clontech). First, the coding sequences of *CLF* and *FT* were cloned into *pGADT7* and *pGBKT7*, respectively. Paired plasmids were introduced into the yeast strain AH109; subsequently, the transformed yeast cells were grown on a solid synthetic medium without leucine (L), tryptophan (W), histidine (H), and adenine (A) to exam potential protein‐protein interaction.

### 4.5 ChIP Assay

ChIP experiments in Arabidopsis were conducted following previously established protocols [57], with minor changes. In brief, total chromatin was extracted from the seedlings of interest grown in LDs, followed by immunoprecipitation with anti‐HA (Sigma, H9658), anti‐FLAG (Sigma, M8823), anti‐GFP (Abcam, ab290), or rabbit polyclonal anti‐CLF [37]. The immunoprecipitated genomic fragments of interest were quantified by qPCR on an *ABI QuantStudio5 Flex* real‐time PCR system (Applied Biosystems) with iTaq SYBR Green supermix (Biorad). The levels of examined *FT* fragments were normalized to the internal background control *TUBULIN2* (*TUB2*); subsequently, relative fold enrichments were calculated over a background control. Three biological replicates were conducted for each ChIP assay.

ChIP assays in soybean were carried out as previously described with minor modifications [58]. Briefly, approximately 1.0 g of fully expanded trifoliate leaves from 14‐d‐old soybean plants grown in SDs was collected at ZT8, and fixed with 1% formaldehyde with vacuum infiltration. Subsequently, the nuclei were isolated following an established protocol [59], followed by total chromatin extraction and subsequent fragmentation by Bioruptor (Diagenode). Immunoprecipitations were carried out with anti‐FLAG beads (Sigma, M8823). Genomic DNA fragments were quantified by qPCR, and subsequently normalized to the internal background control *GmACT11*. Relative fold enrichments were calculated over a background control. Values are means ± s.d. of three biological replicates. Primer sequences are described in Table S1.

### 4.6 Chromosome Conformation Capture (3C) Experiment

3C assays were conducted as previously described with minor modifications [11, 46, 59]. Briefly, nuclei were extracted from about 2‐g seedlings cross‐linked in 2% formaldehyde, and lysed by 0.15% SDS. Next, chromatin‐bound DNA was digested by *Dpn II* (NEB) at 37°C overnight, followed by ligation with T4 DNA ligase (NEB) at 16°C for 6 h. After reversing crosslinks, the ligated DNA was purified by phenol‐chloroform extraction, followed by ethanol precipitation.

Quantitative PCR was conducted to determine relative interaction frequencies between paired regions. The CORE‐bearing region near the transcription start site serves as the anchor segment, and a primer from this region is paired with various primers located at distal *FT* promoter regions for the qPCR analysis. An *FT* region lacking a Dpn II cutting site was used as a loading control, to normalize the differences in DNA concentrations from different samples. Additionally, primer efficiencies were determined using a control template that contained equal amounts of all possible ligation products derived from a 9.1‐kb *FT* fragment that had been digested with Dpn II. Relative contact or interaction frequencies were calculated by normalizing the level of an amplified fragment from WT or *FTproΔC* over that from the control template. Primers are listed in Table S1.

### 4.7 Histological Staining of β‐Glucuronidase Activity

GUS activity staining was conducted as previously described [60]. Briefly, transgenic seedlings (T_1_) grown in LDs were harvested at ZT12, and stained in a 1.5 mM X‐Gluc (5‐bromo‐4‐chloro‐3‐indolyl‐β‐D‐glucuronic acid) solution at 37°C for 12 h, followed by de‐chlorophylling with 70% ethanol. Stained seedlings were examined under a Leica dissecting microscope.

### 4.8 Biomass and Seed Yield Assay

The whole plant biomass assay was conducted as previously described with minor adjustments [45]. In brief, plants were cultivated in soil until a main stem of 1 cm in height had developed after bolting. Fresh weights of 10 seedlings for each line were measured, followed by a full dehydration at 65°C for 24 h and dry weight measurement.

Seed yields per plant were measured as follow. Plants were grown in soil until no flowers were produced, followed by withholding water for approximately 4 weeks to ensure complete drying of the inflorescences. Subsequently, seeds were harvested from individual plants, and thoroughly dried, and the total dry seed mass per plant was measured.

### 4.9 Cryosectioning and Confocal Imaging

Samples were cryo‐sectioned and examined with a confocal microscope as described previously with minor modifications [49]. Briefly, leaves or shoot apical regions from seedlings grown in LDs, were fixed in 4% paraformaldehyde (in Phosphate‐Buffered Saline /PBS, pH 6.9) at room temperature for 1 h after vacuum infiltration. Next, the samples were washed by PBS and incubated in 30% sucrose (in PBS) overnight, followed by embedding in Tissue Tek O.C.T. compound (Sakura Finetek) and sectioned to 20‐µm thickness by a Leica CM 1950 sliding microtome at ‐20°C. Slides were dried in a 42°C oven and treated briefly with the 1:1 methanol/acetone mixture, and then were mounted with PBS and imaged under a Zeiss LSM900 confocal microscope.

### 4.10 Soybean Transformation

The recombinant *35S_pro_‐GmFT4:FLAG* was introduced into the *Agrobacterium tumefaciens* strain *EHA105* and subsequently transformed the soybean cultivar *Williams 82* using the cotyledonary node method as previously described, utilizing the *bar* (bialaphos resistance) gene as a selectable marker in plants with glufosinate as the selection agent [61]. Briefly, soybean seeds were surface‐sterilized in a desiccator, by exposure to chlorine gas produced from a mixture of hydrochloric acid and sodium hypochlorite. Following overnight sterilization, the seeds were placed on a germination medium plate (24°C for 1 day). After seed germination, the apical shoot/bud was removed, and 3 to 5 incisions (0.5 mm deep and 3–4 mm long) were made at the junction of cotyledon with hypocotyl. The explants were then inoculated with a freshly‐prepared *EHA105* culture, followed by co‐cultivation, shoot induction and growth, and rooting. Subsequently, the bar‐resistant seedlings were transplanted into soil, and the seeds from verified transgenic plants were harvested.

### 4.11 Statistical Analysis

Two‐tailed *Student's t* test and one‐way ANOVA (Analysis of Variance) with the Holm‐Sidak method were conducted using GraphPad Prism (v9.5.1). All discrete leaf number data were log‐transformed for statistical analysis.

## Conflicts of Interest

The authors declare no conflicts of interest.

## Supporting information




**Supporting File**: advs76494‐sup‐0001‐SuppMat.pdf.

## Data Availability

The data that support the findings of this study are available in the article and its Supporting Information.
