# Supplementary material for: Auto‐Downregulation of the Florigen FT Production Prevents Precocious Flowering in Plants
Source: Adv Sci (Weinh). 2026 Jul 13;13(55):e22307. doi: 10.1002/advs.202522307 (PMC13360168; doi:10.1002/advs.202522307)
Supplement: Supplementary file 1 [file ADVS-13-e22307-s001.pdf]

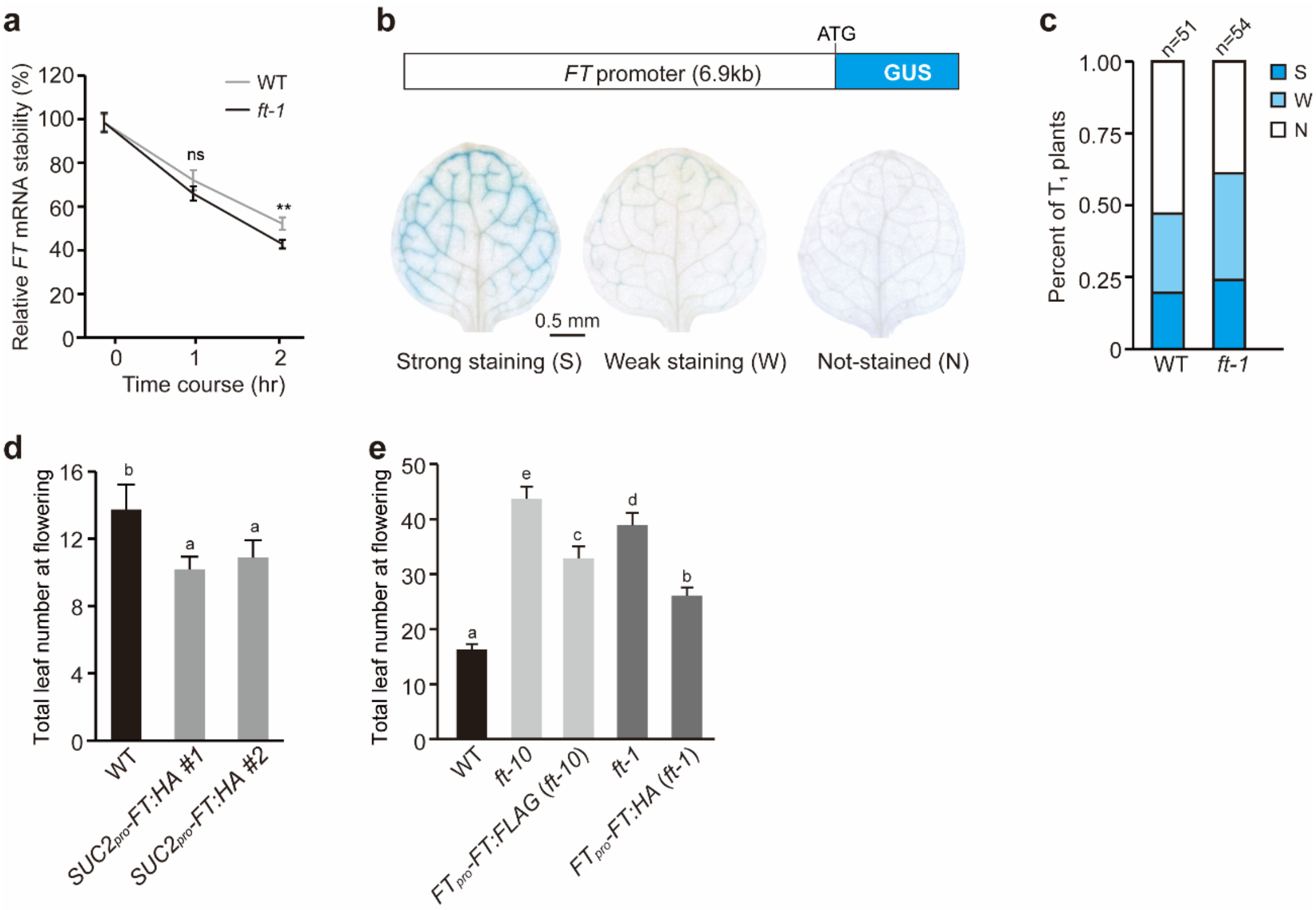

Figure S1. Characterization of auto-repression of *FT* expression. a) *FT* mRNA decay in WT and *ft-1* seedlings in LDs. Seedlings were treated with 0.6 mM cordycepin in an incubation buffer (1 mM PIPES pH 6.25, 1 mM sodium citrate, 1 mM potassium chloride and 15 mM sucrose). Transcript levels were quantified by qPCR and first normalized to the internal control 18S rRNA, and relative levels to the initial time point were calculated. Values are means ± s.d. of three biological replicates. Two-tailed *t* test was conducted to assess mean differences at indicated time points (** $p < 0.01$ and ns for not significant). **b)** Classification of GUS staining in transgenic seedlings (T_1 generation) expressing *FTpro-GUS* (in WT) under LD conditions. Seedlings were classified into three groups based on the staining intensity in leaves: strongly stained (S), weakly stained (W), and not stained (N). **c)** Analysis of GUS staining in leaves of the indicated FT_{pro}-*GUS* transgenic plants (T_1 generation) grown in LDs. The percentages of seedlings exhibiting different levels of staining were calculated (n for total number of plants scored in each line). **d** and **e)** Flowering times of *SUC2pro-FT:HA* lines (**d**) and *ft* rescue lines (**e**) grown in LDs. Total number of leaves formed prior to flowering was scored (15-18 plants per line), and bars for s.d. Letters indicate statistically significant differences (one-way ANOVA; $p<0.01$).

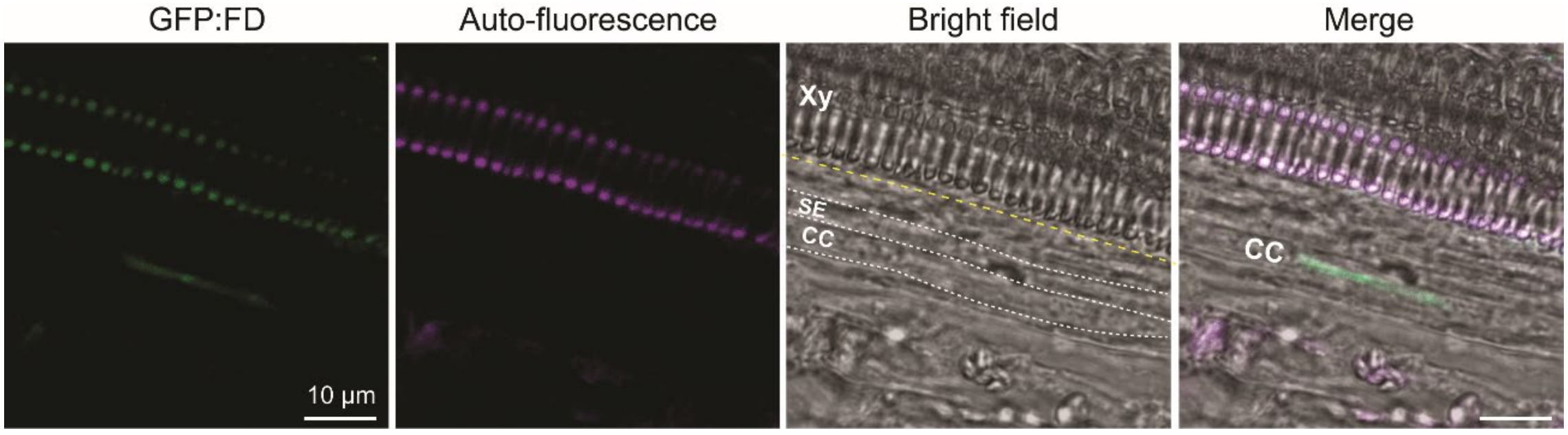

Figure S2. FD is localized in the companion cells of phloem. Confocal imaging of the GFP:FD localization in major leaf veins (longitudinal sections). Xy (xylem), SE (sieve elements), and CC (companion cells) are indicated by broken lines. Note that purple color indicates autofluorescence. Scale bars, 10 μm.

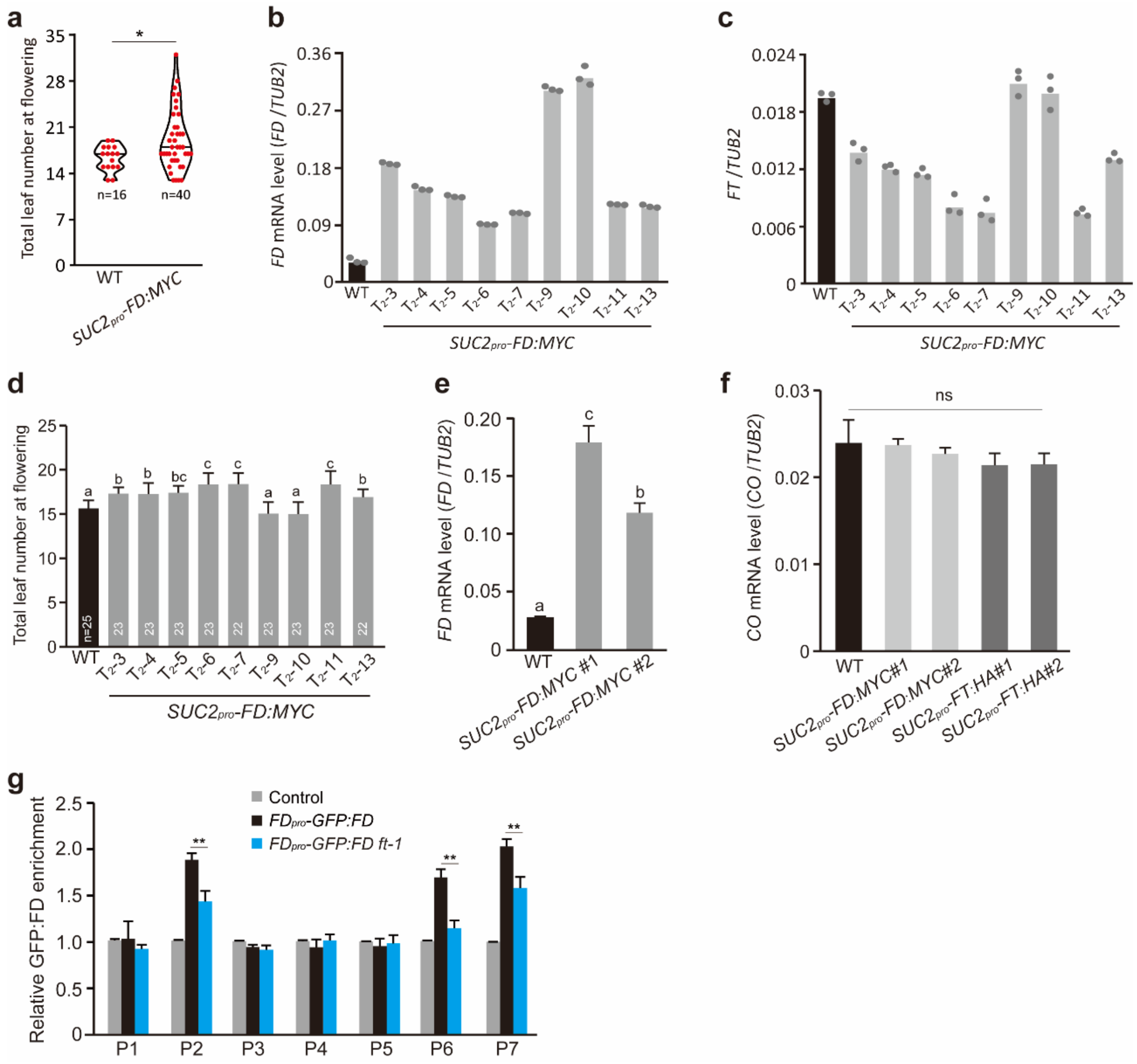

Figure S3. Characterization of *FD*-mediated *FT* repression in leaf veins. a) Flowering times of the T_1 lines of *SUC2*$_{pro}$*-FD:MYC* in comparison with WT (Col-0). Total number of leaves formed prior to flowering was scored. Data points are plotted on violin plots with medians indicated by solid lines. **b,c)** *FD* (**b**) and *FT* mRNA (**c**) expression in the first pair of rosette leaves of WT and single-locus T_2 lines of *SUC2*$_{pro}$*-FD:MYC* grown in LDs (14-d-old). Samples were harvested at ZT12, and transcript levels were quantified by RT-qPCR and normalized directly to *TUB2*. Values are means ± s.d. of three technical replicates. **d)** Flowering times of individual T_2 lines of *SUC2*$_{pro}$*-FD:MYC*. Total number of leaves formed prior to flowering was scored (22-25 plants per line were scored, as indicated). **e)** *FD* mRNA expression in rosette leaves of homozygous single-locus T_3 lines of *SUC2*$_{pro}$*-FD:MYC* grown in LDs. Values are means ± s.d. of three biological replicates. **f)** *CO* expression in rosette leaves of WT, *SUC2*$_{pro}$*-FT:HA* lines and *SUC2*$_{pro}$*-FD:MYC* lines under LDs (at ZT16). *CO* transcripts were quantified by RT-qPCR and normalized to *TUB2*. Values are means ± s.d. of three

biological replicates. **g)** FT is partly required for GFP:FD binding to *FT* promoter regions, as revealed by ChIP-qPCR with anti-GFP. Relative GFP:FD fold enrichments in each *FT* promoter region over the background control (WT IP with anti-GFP) are presented. Samples were harvested at ZT16 (LDs). Values are means ± s.d. of three biological replicates. One-way ANOVA was conducted in (**d-f**), with letters to indicate statistically significant differences (ns for not significant), whereas in (**a**, **g**) two-tailed *t* tests were carried out (* $p<0.05$ and ** $p<0.01$).

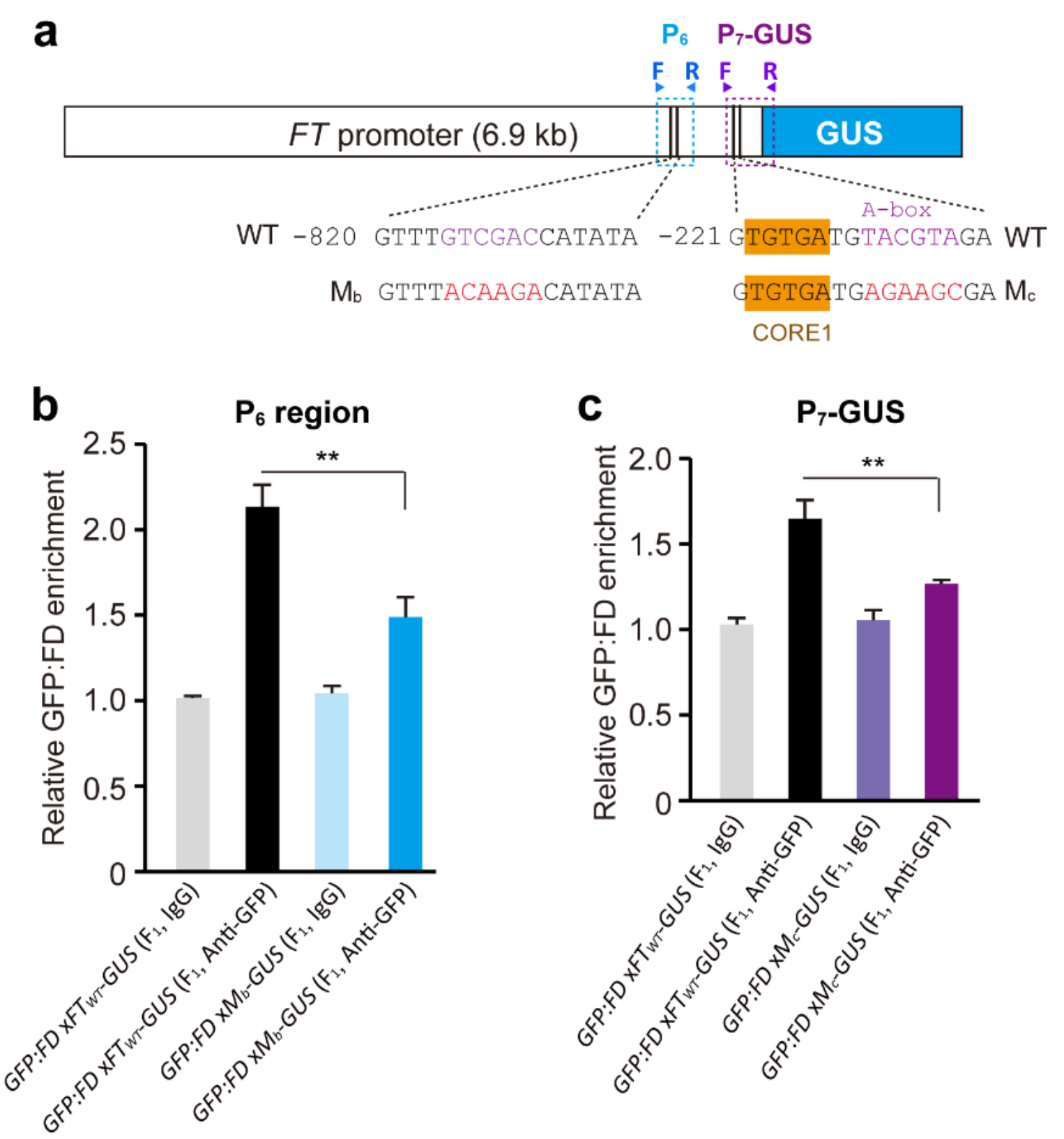

Figure S4. FD binds both the GTCGAC motif and A-box in *FT* promoter. a) Illustration of *FT-GUS* with M_b (a mutated GTCGAC motif in the P_6 region) or M_c (a mutated A-box in the P_7 region) as shown in Figure 4a. F and R indicate the paired primers used in ChIP-qPCR. **b)** ChIP-qPCR analysis of GFP:FD enrichment in the P_6 regions of *FT-GUS* and the endogenous *FT*. The *GFP-FD* line was crossed to FT_{wt}-*GUS* and FT_{Mb}-*GUS* lines (in the WT background). Total chromatin was extracted from the F_1 seedlings (sampled at ZT16), followed by immunoprecipitation using anti-GFP or anti-IgG (background control). Relative GFP:FD fold enrichments at the P_6 and/or P_6 (M_b) region over the background control are shown. Notably, the P_6 fragments from the F_1 seedlings of *GFP:FD* x FT_{Mb}-*GUS* are composed of P_6 (M_b) (from FT_{Mb}-*GUS*) and P_6 (from the native *FT*); hence, the moderate enrichment of GFP:FD in these F_1 seedlings conceptually is contributed largely by the P_6 fragments from the native *FT*. **c)** ChIP-qPCR analysis of GFP:FD enrichment in the P_7 or P7 (Mc) regions of *FT-GUS*. Note that only the fragments of P_7 from FT_{wt}-*GUS* or P7 (Mc) from FT_{Mc}-*GUS* were amplified in qPCR. **b, c)** Values are means ± s.d. of three biological replicates. Two-tailed *t* tests were conducted to evaluate statistically significant differences (** p<0.01).

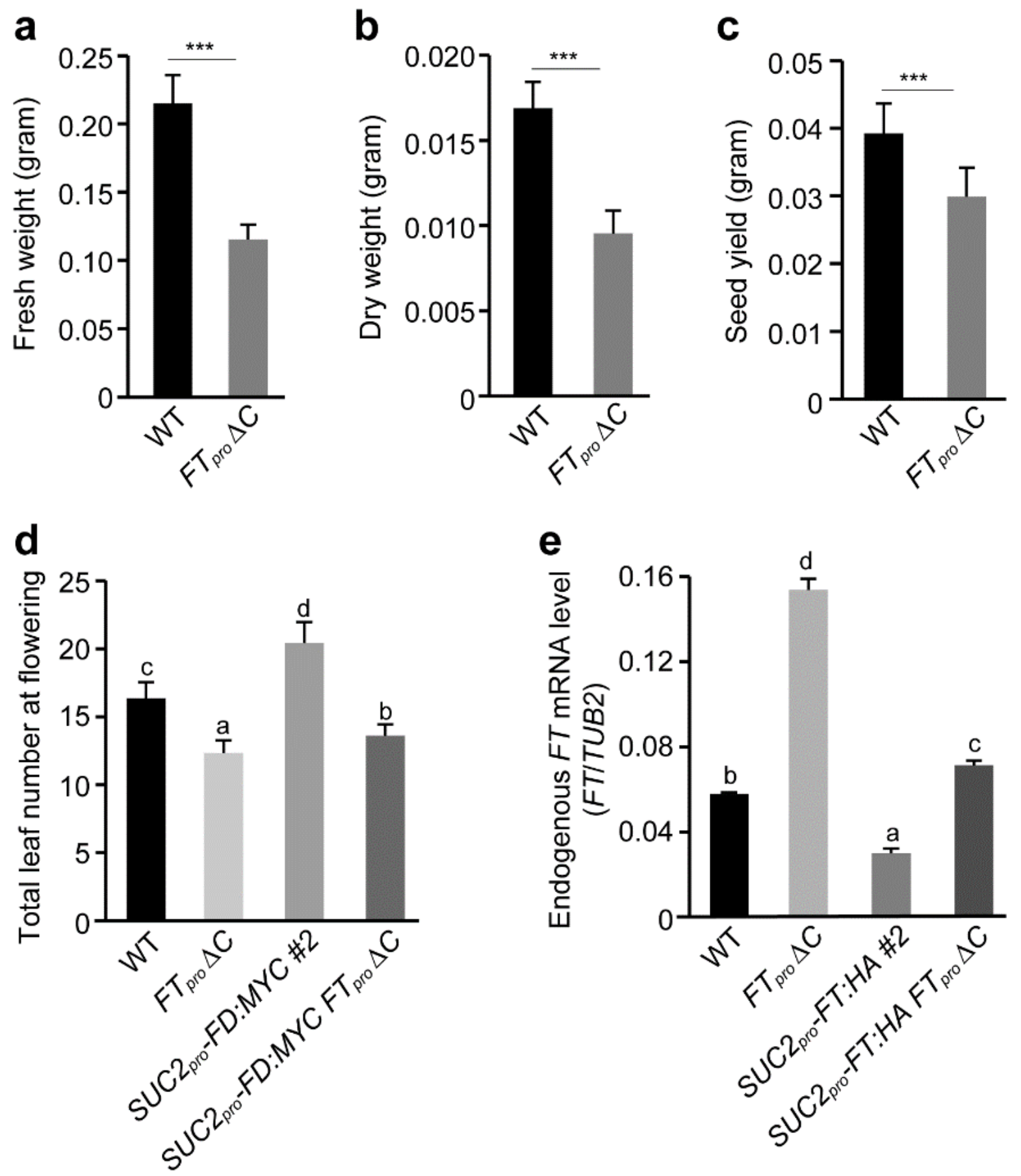

Figure S5. Characterizations of $FT_{pro}\Delta C$. a-c) $FT_{pro}\Delta C$ exhibits a great decrease in biomass under normal growth conditions in LDs. Fresh weight (**a**), dry weight (**b**) and seed yield (**c**) are measured. A total of 10 plants were scored for each line. **d)** FD-mediated delay in flowering is largely suppressed by *FTpro ΔC*. Total number of leaves formed prior to flowering was scored for each line (30 plants per line grown in LDs). Bars for s.d. and letters denote statistically-significant differences (one-way ANOVA, p<0.01). **e)** Downregulation of the endogenous *FT* expression in $SUC2_{pro}$-*FT:HA* lines is partly rescued by $FT_{pro}\Delta C$. Seedlings were grown in LDs and harvested at ZT16. **a-c**, **e)** Values are means ± s.d. of three biological replicates. Two-tailed *t* tests were conducted in (**a-c**) to evaluate statistically significant differences (*** p<0.001), while one-way ANOVA was conducted in (**e**), with letters to indicate statistically-distinct means (p<0.01).

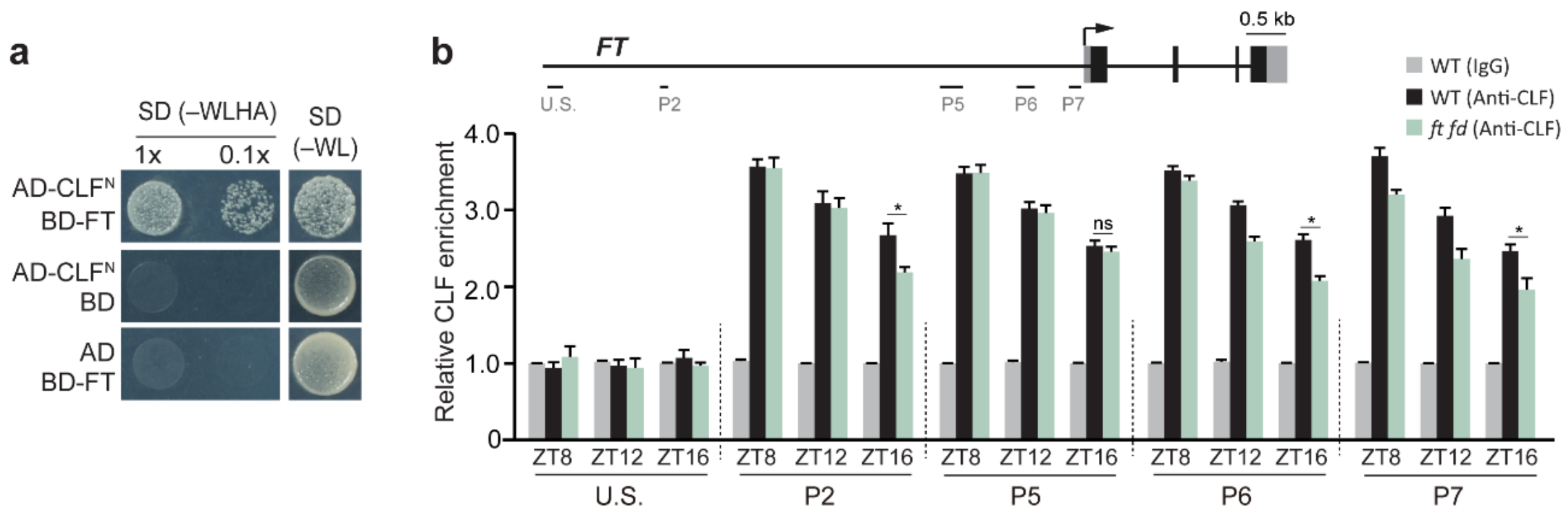

Figure S6. FT interacts with CLF and is partly involved in CLF binding *FT* chromatin. a) FT interacted with an N-terminal region of the CLF protein (aa 1 to 556) in yeast cells. Yeast was grown on a stringent selective medium lacking of tryptophan (W), leucine (L), histidine (H) and adenine (A), and cells grown on the SD media lacking of W and L serve as growth control. **b)** ChIP analysis of dynamic CLF enrichment on *FT* chromatin in WT and *ft fd* seedlings grown in LDs. Levels of *FT* fragments immunoprecipitated by rabbit polyclonal anti-CLF were quantified by qPCR and normalized to the internal control *TUB2*. Shown are relative fold enrichments of CLF in WT and *ft fd* over a background control (the immunoprecipitation with IgG). Values are means ± s.d. of three biological replicates. Statistical significance of indicated mean differences are assessed using two-tailed *t* test (* $p < 0.05$, and ns for not significant).

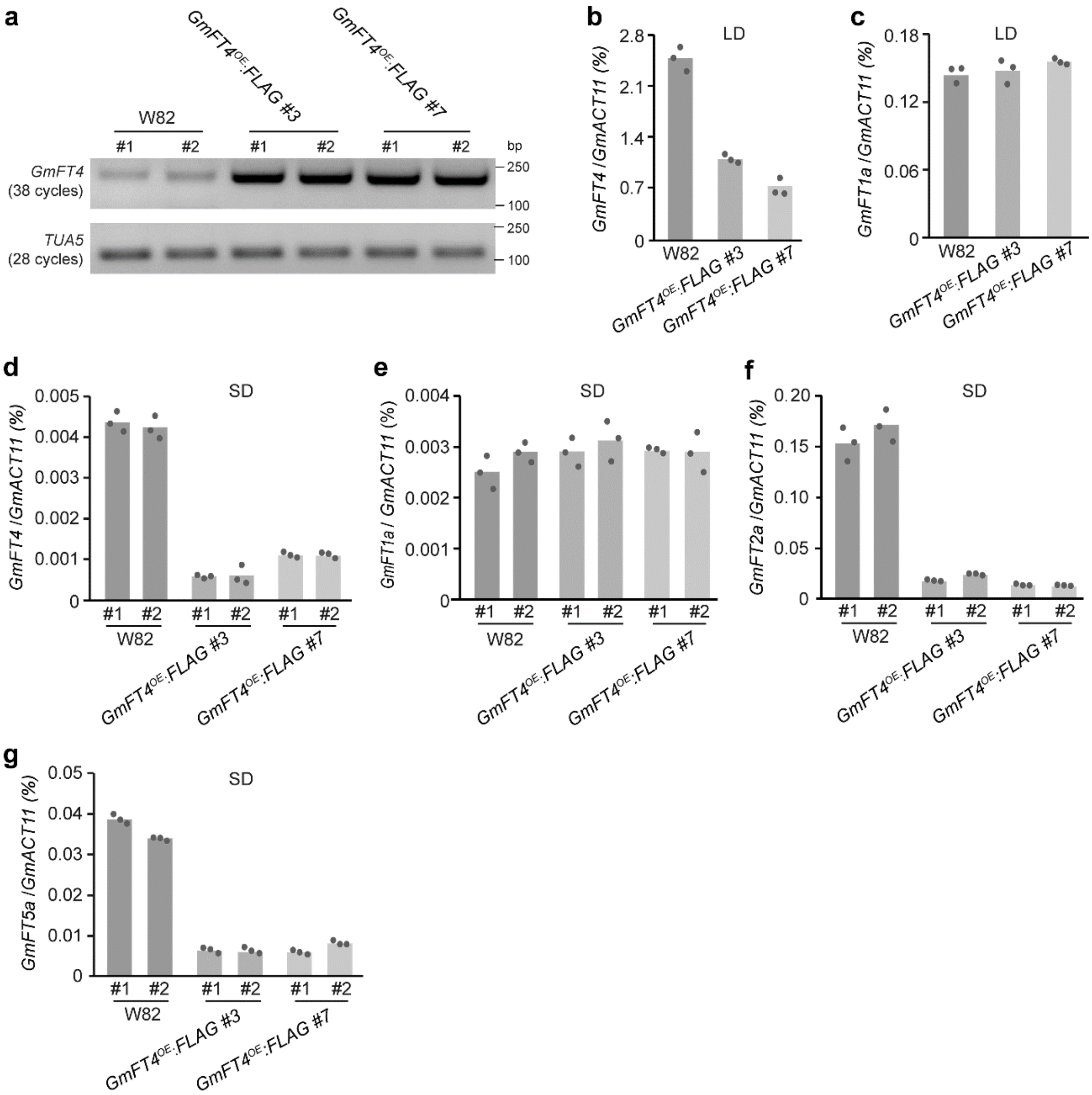

Figure S7. Repression of *FT-like* genes by *GmFT4* overexpression in soybean. a) Analysis of *GmFT4:FLAG* overexpression in leaves of the indicated transgenic soybean lines by semiquantitative RT-PCR. Two individual soybean plants from each transgenic line or *Williams 82* (grown in SDs) were examined. The constitutively-expressed *TUBULIN ALPHA5* (*TUA5*) serves as an internal control. W82 for *Williams 82*, the non-transgenic background. **b**,**c)** Transcript levels of endogenous *GmFT4* (**b**) and *GmFT1a* (**c**) in the leaves of W82 and *GmFT4^{OE}:FLAG* lines at 21 DAE under LDs. Leaves from two independent T_3 soybean lines (5 plants per line) were harvested at ZT16 for RNA extraction. Transcript levels were quantified by RT-qPCR and normalized to the constitutively expressed *GmACT11*. Values are means ± s.d. of three technical replicates. **d-g)** Transcript levels of endogenous *GmFT4* (**d**), *GmFT1a* (**e**)

GmFT2a (**f**), and *GmFT5a* (**g**), and in the leaves of W82 and *GmFT4*^{OE}*:FLAG* lines at 14 DAE under SDs. Samples were harvested at ZT8. Two individual soybean plants from each transgenic line or W82 were examined, and data points of three technical replicates for each sample are overlaid on bar graphs.

Table S1. List of primers used in this study

Experiment	Amplified region	Sequence (5'-3')
RT-PCR	*GmFT4*	F: CTACAGGGGCAACCACTGGAGAAGA
		R: TCTCCTTCGTCCACCCCAACCA
	GmTUA5	F: TGCCACCATCAAGACTAAGAGG
		R: ACCACCAGGAACAACAGAAGG
RT-qPCR	*FT* (CDS)	F: GACCTCAGGAACTTCTATACTTTGGTTATG
		R: CTGTTTGCCTGCCAAGCTG
	FT (*3' UTR*)	F: ACTGGAACAACCTTTGGCAATGAGA
		R: CAATTGGTTATAAAGGAAGAAGCCAT
	FD	F: GCAAGACTCAAGAGACAACAAG
		R: CAAAATGGAGCTGTGGAAGAC
	FT:HA	F: TCCCACTGCAGGAATTCATCGT
		R: TCGTATGGGTACTCGAGTGC
	CO	F: CACTACAACGACAATGGTTCC
		R: GGTCAGGTTGTTGCTCTACTG
	GUS	F: CTCCTACCGTACCTCGCATTAC
		R: ACGCGCTATCAGCTCTTTAATC
	TUB2	F: GCCTTGTACGATATTTGCTTCAGGAC
		R: CGGAGGTCAGAGTTGAGTTGAC
	UBQ10	F: GATCTTTGCCGGAAAACAATTGGAGGA
		R: CGACTTGTCATTAGAAAGAAAGAGATAAT
	GmFT1a	F: TGCAACTACAGGGGCAAACT
		R: GTTGAAGTACATGGCCGCTA
	GmFT2a	F: GGATTGCCAGTTGCTGCTGT
		R: GAGTGTGGGAGATTGCCAAT
	GmFT5a	F: GCCTTACTCCAGCTGATACT
		R: GGCATGCTCTAGCATTGCAA
	GmFT4 (*3 'UTR*)	F: CTACAGGGGCAACCACTGGAGAAGA
		R: GGACAACGTGCGCAGTGTGA
	GmACT11	F: CGGTGGTTCTATCTTGGCATC
		R: GTCTTTCGCTTCAATAACCCTA
ChIP-qPCR	*FT U.S.*	F: GCGGCATTGTACTAAACGAAA
		R: GCCACCAAATCTTATCACCCT
	FT P1	F: ACGTAAATTATGACACTAAATGGTGC
		R: GTGTTGACGTTGAGTTTGTGATCTT

ChIP-qPCR	*FT P2*	F: ACGTTGATGATAGTGAAGTGA<br>R: ACGCAACCAAGTAGAGACGT
	FT P2a	F: CGTTGATGATAGTGAAGTGAGACATCTTGG<br>R: AAAACGTTTGGAAATAGGAAGTATGTAAAAACGAA
	FT P3	F: TGTAGCACAAGATCTTTTGGAGAACT<br>R: TGATTTCATTTTGTCGAGATTTAGAAG
	FT P4	F: ATCCACTTGCCAATCTTCGTAAT<br>R: TCATTGGTGTAATGACCATGATAAGA
	FT P5	F: GTGGCGGACAATCCATCTATCTC<br>R: GATCGACCATTGATAATCGAAATCGCA
	FT P6	F: GCAGATATCTTGTACTTAATTCATTTTG<br>R: GCCTTTTCTTGTAGTTATACCAGGTA
	FT P7	F: GTGTGGTGGGTTTGGAATACC<br>R: ACTCGGGTCGGTGAAATCAT
	FT E1	F: ATAGTAAGCAGAGTTGTTGGAGACG<br>R: CAATCTCAACTCTTGGCTTGTTTTG
	FT I1	F: GCCCCACGCTTTCCTTTTCTCTGTT<br>R: CACTTATGCAAGAAGTTGGTGGAAA
	FT I2	F: TCTCCATTGGTTTGTGCACTAACTC<br>R: TCGAAGTTGATTTTATATGTCTCCTTC
	FT (*P_7*)-*GUS*	F: CCAGTGTATTAGTGTGGTGGGT<br>R: GTTTTTTGATTTCACGGGTTGGGG
	TUB2	F: ATCCGTGAAGAGTACCCAGAT<br>R: AAGAACCATGCACTCATCAGC
	GmFT2a P1	F: ACTCCTTCTGATCTCAATTGTAAG<br>R: AACTAAATTTTTCTTATTGAGATTATC
	GmFT2a P2	F: CAACAAACAAGATGTATGGTTTTCA<br>R: TCATTCAATGGAATTATAAATCGTTT
	GmFT2a P3	F: TTCTAAAACAGAAAGAAACTCATTATAT<br>R: TAAACCTTTTACTTTGTGATTTAATA
	GmFT2a P4	F: GATAGATATTCGTTCACCATATAGAG<br>R: AATTAAGAATCATTGATTGACTTTC
	GmFT2a P5	F: ACTCAAGTGTTGCCAATTAATTGAC<br>R: CATCTTCTCATTTTCTCTCTTCCATT
	GmFT2a I1	F: CTCTTTTAACTTTGTATTCAAACAATC<br>R: GTACTTGACCTTCCCTTAAACACA
	GmFT5a P1	F: ATGTCCCTCATATTTGTAATAAAGCC<br>R: AATGATGATGCAAACACATTATTCAT

ChIP-qPCR	*GmFT5a P2*	F: ATTAGGATTCCAACCAAACACAACT<br>R: AGATAAACCCTAAACTATTTTTGGAT
	GmFT5a P3	F: TGTAGATACCACGAAATAGAACTTACAT<br>R: AAAGAGATAATCTGTGTCCATATTCG
	GmFT5a 3'UTR	F: ATAATTTAGAGAGAAGGTACCATCTT<br>R: TAGCCAGAAAAGAAAGGACATCATAA
	GmFT4 P1	F: GATAAGTGTTTTAGTTGCCAAGAATG<br>R: GATATTGATAACTTCCATTGTCCATG
	GmFT4 P2	F: ATTTGTCTTGCTCTGTTTGTGCA<br>R: GTCCCTGAGTCCTGAATCCTAAAT
	GmFT4 P3	F: GTGGCTGTTGTAGAATAGAGGGA<br>R: AACTAAAGAACAAAATTAAAATAGCGA
	GmFT4 P4	F: AAGTCTCCTTGTTAAATTGCCTAATC<br>R: ATTAGAGCCTTTTCCTTTCTCATTG
	GmFT4 P5	F: AGTTCTGCCTCAAGTTTGTCTATAAAG<br>R: AGGCCTTCAAATATGACAAATCAC
	GmFT4 P6	F: GTCAAGGTGGCTCAATGAAGAAC<br>R: CACCACTAATCCAATTTTGATGG
	GmFT4 P7	F: TTTTGCATGACAAACACTAACCAC<br>R: GAAAAAGAGTCAGAAGAACGGGA
	GmFT4 P8	F: CTTAGAGGAAAAGGAAAACCAGAAT<br>R: GAGAAACAGAACTCGTGAAGGGA
	GmACT11	F: CGGTGGTTCTATCTTGGCATC<br>R: GTCTTTCGCTTCAATAACCCTA
3C-qPCR	*FT I*	R: GACCATCCTGTGTTGACGTTGAGTTTG
	FT II	R: CTAATGTTGGCCAAGATGTCTCAC
	FT III	F: GGATTGACACATATCTCTTATATACTAGTG
	FT anchor	F: GAAGCAGAAACAAAAACAAGTAAAACAG
	FT loading CK	F: GGATTTTTCTTTGTTCCTCCTACCT<br>R: CTGGCTTGAATATCAGAATATCCTTAT
