# Supplementary material for: Auto‐Downregulation of the Florigen FT Production Prevents Precocious Flowering in Plants
Source: Adv Sci (Weinh). 2026 Jul 13:e22307. Online ahead of print. doi: 10.1002/advs.202522307 (PMC13360168; doi:10.1002/advs.202522307)
Supplement: Supplementary file 1 — Supporting File: advs76494‐sup‐0001‐SuppMat.pdf. [file ADVS-9999-e22307-s001.pdf]

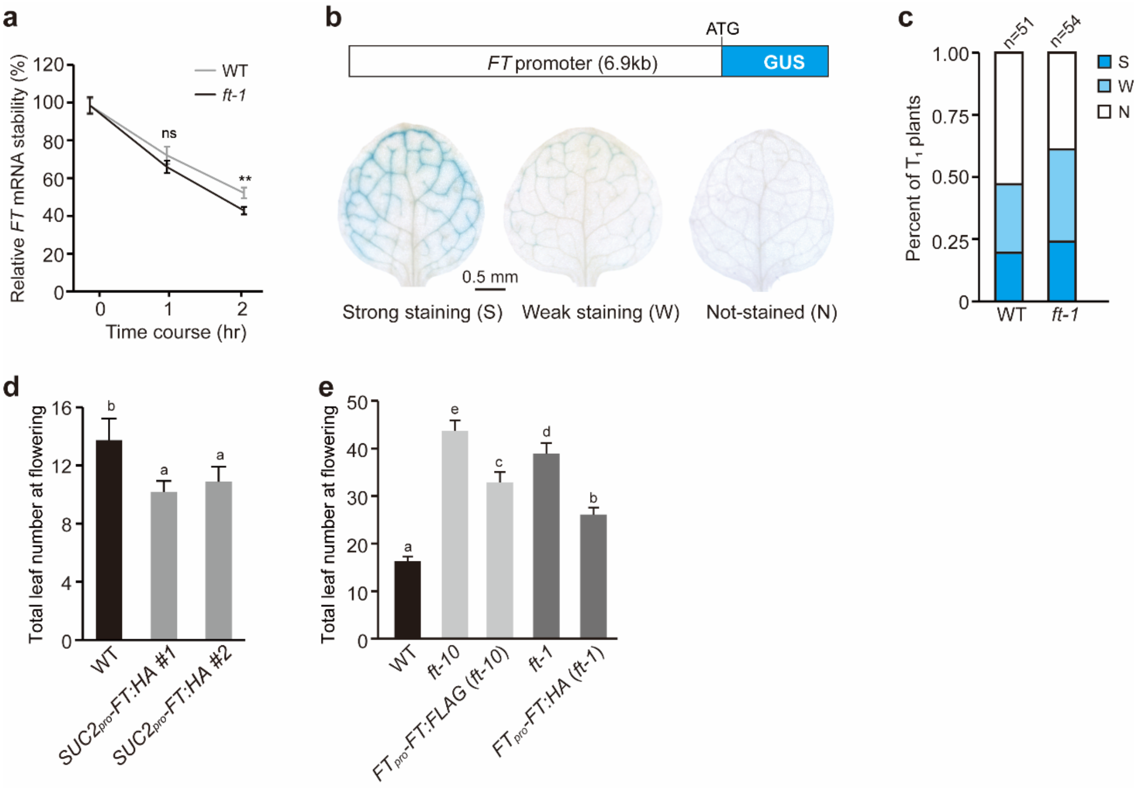


**Figure S1. Characterization of auto-repression of *FT* expression. a)** *FT* mRNA decay in WT and *ft-1* seedlings in LDs. Seedlings were treated with 0.6 mM cordycepin in an incubation buffer (1 mM PIPES pH 6.25, 1 mM sodium citrate, 1 mM potassium chloride and 15 mM sucrose). Transcript levels were quantified by qPCR and first normalized to the internal control 18S rRNA, and relative levels to the initial time point were calculated. Values are means ± s.d. of three biological replicates. Two-tailed *t* test was conducted to assess mean differences at indicated time points (** $p< 0.01$ and ns for not significant). **b)** Classification of GUS staining in transgenic seedlings ($T_1$ generation) expressing *FTpro-GUS* (in WT) under LD conditions. Seedlings were classified into three groups based on the staining intensity in leaves: strongly stained (S), weakly stained (W), and not stained (N). **c)** Analysis of GUS staining in leaves of the indicated $FT_{pro}$-*GUS* transgenic plants ($T_1$ generation) grown in LDs. The percentages of seedlings exhibiting different levels of staining were calculated (n for total number of plants scored in each line). **d** and **e)** Flowering times of *SUC2pro-FT:HA* lines (**d**) and *ft* rescue lines (**e**) grown in LDs. Total number of leaves formed prior to flowering was scored (15-18 plants per line), and bars for s.d. Letters indicate statistically significant differences (one-way ANOVA; $p<0.01$).

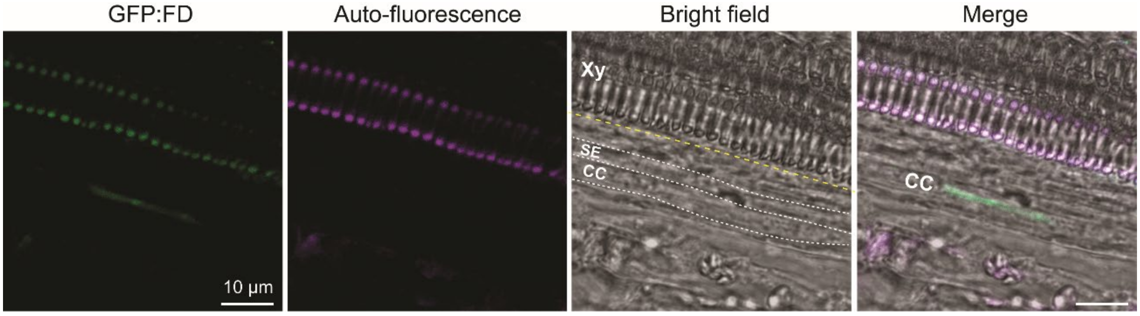


**Figure S2. FD is localized in the companion cells of phloem.** Confocal imaging of the GFP:FD localization in major leaf veins (longitudinal sections). Xy (xylem), SE (sieve elements), and CC (companion cells) are indicated by broken lines. Note that purple color indicates autofluorescence. Scale bars, 10 μm.

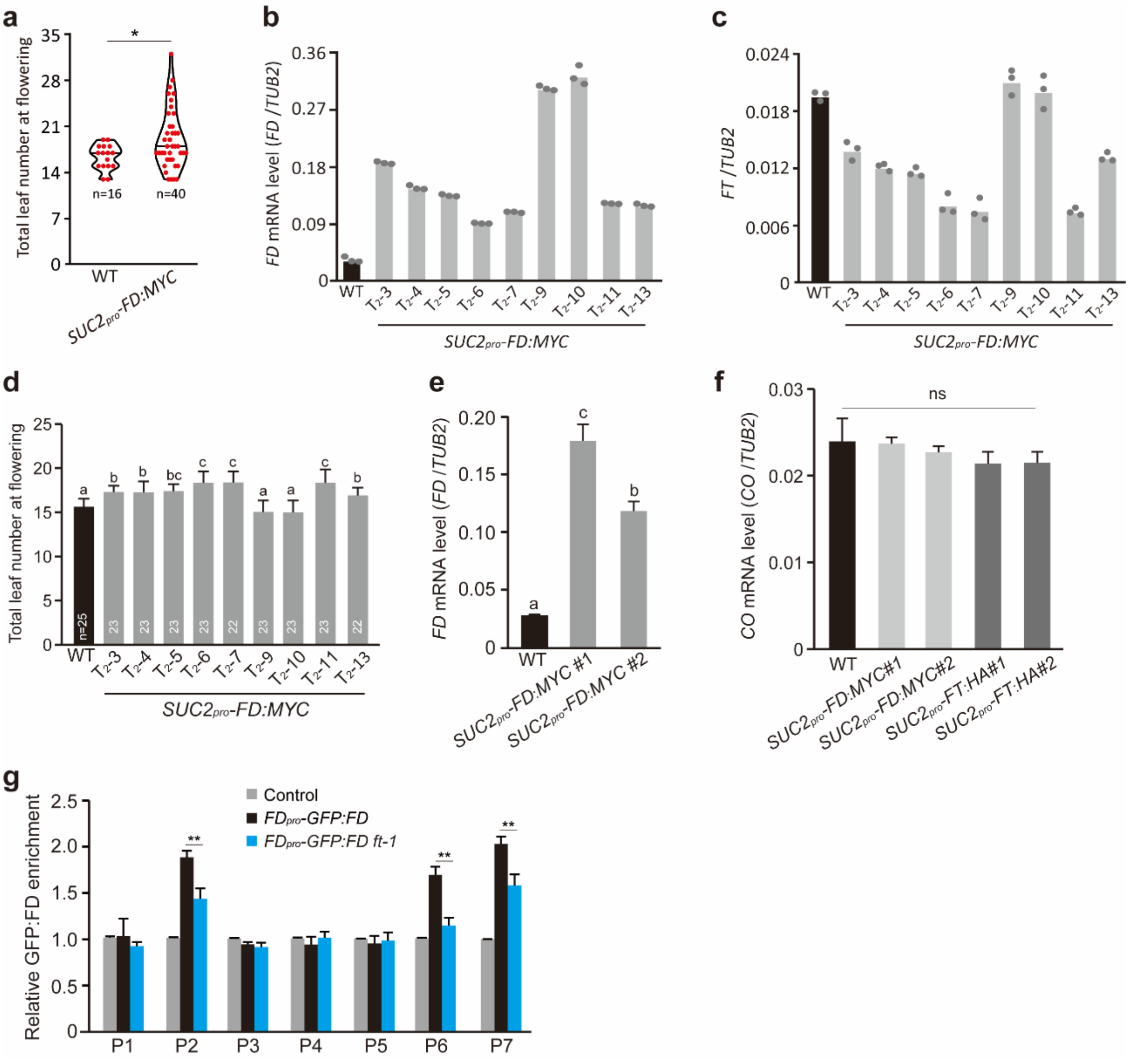


**Figure S3. Characterization of *FD*-mediated *FT* repression in leaf veins. a)** Flowering times of the $T_1$ lines of *SUC2$_{pro}$-FD:MYC* in comparison with WT (Col-0). Total number of leaves formed prior to flowering was scored. Data points are plotted on violin plots with medians indicated by solid lines. **b**,**c)** *FD* (**b**) and *FT* mRNA (**c**) expression in the first pair of rosette leaves of WT and single-locus $T_2$ lines of *SUC2$_{pro}$-FD:MYC* grown in LDs (14-d-old). Samples were harvested at ZT12, and transcript levels were quantified by RT-qPCR and normalized directly to *TUB2*. Values are means ± s.d. of three technical replicates. **d)** Flowering times of individual $T_2$ lines of *SUC2$_{pro}$-FD:MYC*. Total number of leaves formed prior to flowering was scored (22-25 plants per line were scored, as indicated). **e)** *FD* mRNA expression in rosette leaves of homozygous single-locus $T_3$ lines of *SUC2$_{pro}$-FD:MYC* grown in LDs. Values are means ± s.d. of three biological replicates. **f)** *CO* expression in rosette leaves of WT, *SUC2$_{pro}$-FT:HA* lines and *SUC2$_{pro}$-FD:MYC* lines under LDs (at ZT16). *CO* transcripts were quantified by RT-qPCR and normalized to *TUB2*. Values are means ± s.d. of three

biological replicates. **g)** FT is partly required for GFP:FD binding to *FT* promoter regions, as revealed by ChIP-qPCR with anti-GFP. Relative GFP:FD fold enrichments in each *FT* promoter region over the background control (WT IP with anti-GFP) are presented. Samples were harvested at ZT16 (LDs). Values are means ± s.d. of three biological replicates. One-way ANOVA was conducted in (**d-f**), with letters to indicate statistically significant differences (ns for not significant), whereas in (**a**, **g**) two-tailed *t* tests were carried out (* $p<0.05$ and ** $p<0.01$).

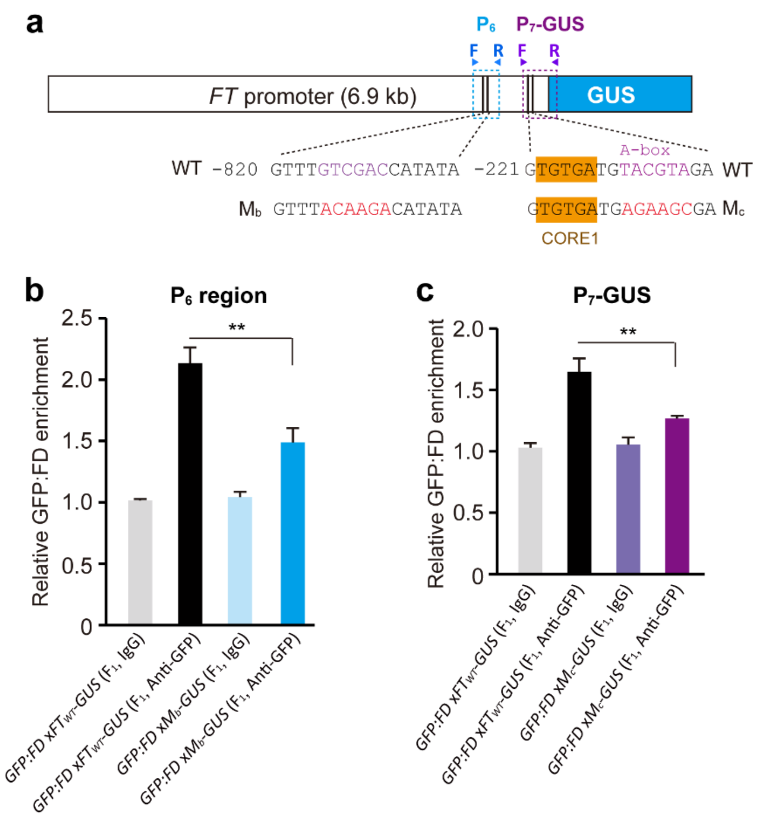


**Figure S4. FD binds both the GTCGAC motif and A-box in *FT* promoter. a)** Illustration of *FT-GUS* with $M_b$ (a mutated GTCGAC motif in the $P_6$ region) or $M_c$ (a mutated A-box in the $P_7$ region) as shown in Figure 4a. F and R indicate the paired primers used in ChIP-qPCR. **b)** ChIP-qPCR analysis of GFP:FD enrichment in the $P_6$ regions of *FT-GUS* and the endogenous *FT*. The *GFP-FD* line was crossed to $FT_{wt}$-*GUS* and $FT_{Mb}$-*GUS* lines (in the WT background). Total chromatin was extracted from the $F_1$ seedlings (sampled at ZT16), followed by immunoprecipitation using anti-GFP or anti-IgG (background control). Relative GFP:FD fold enrichments at the $P_6$ and/or $P_6$ ($M_b$) region over the background control are shown. Notably, the $P_6$ fragments from the $F_1$ seedlings of *GFP:FD* x $FT_{Mb}$-*GUS* are composed of $P_6$ ($M_b$) (from $FT_{Mb}$-*GUS*) and $P_6$ (from the native *FT*); hence, the moderate enrichment of GFP:FD in these $F_1$ seedlings conceptually is contributed largely by the $P_6$ fragments from the native *FT*. **c)** ChIP-qPCR analysis of GFP:FD enrichment in the $P_7$ or P7 (Mc) regions of *FT-GUS*. Note that only the fragments of $P_7$ from $FT_{wt}$-*GUS* or P7 (Mc) from $FT_{Mc}$-*GUS* were amplified in qPCR. **b, c)** Values are means ± s.d. of three biological replicates. Two-tailed *t* tests were conducted to evaluate statistically significant differences (** $p$<0.01).

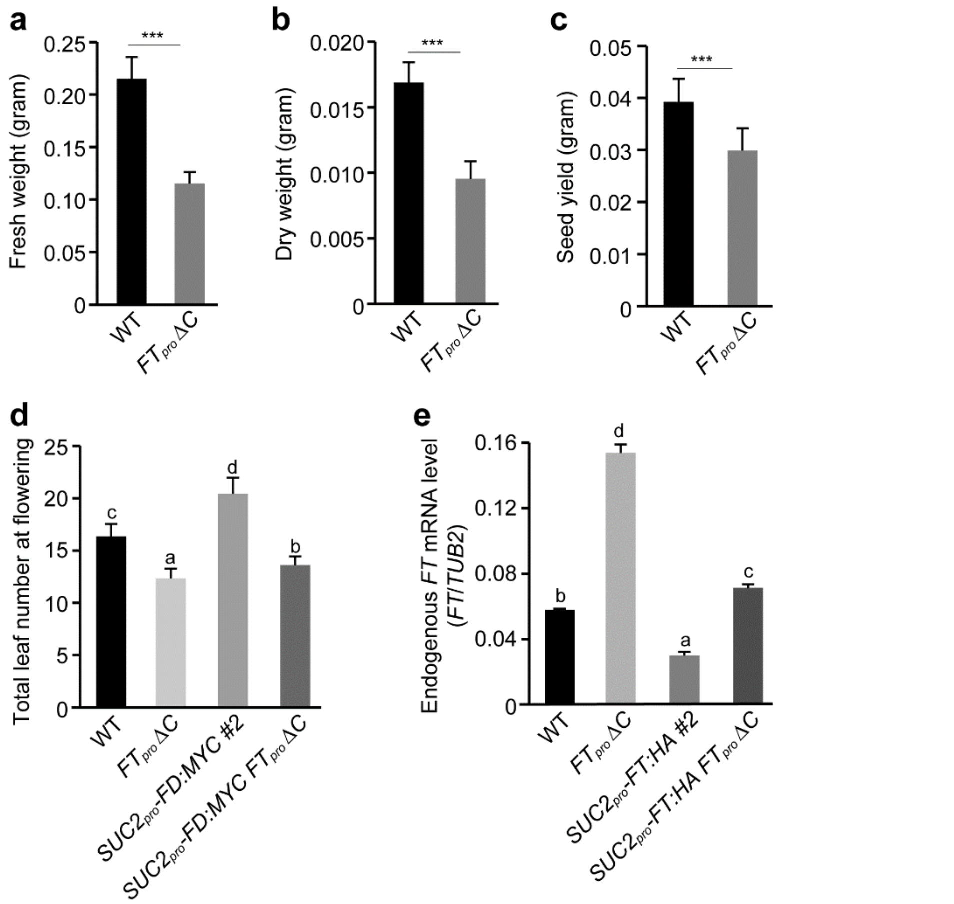


**Figure S5. Characterizations of *FT$_{pro}$ΔC*. a-c)** *FT$_{pro}$ΔC* exhibits a great decrease in biomass under normal growth conditions in LDs. Fresh weight (**a**), dry weight (**b**) and seed yield (**c**) are measured. A total of 10 plants were scored for each line. **d)** FD-mediated delay in flowering is largely suppressed by *FTpro ΔC*. Total number of leaves formed prior to flowering was scored for each line (30 plants per line grown in LDs). Bars for s.d. and letters denote statistically-significant differences (one-way ANOVA, $p$<0.01). **e)** Downregulation of the endogenous *FT* expression in *SUC2$_{pro}$-FT:HA* lines is partly rescued by *FT$_{pro}$ΔC*. Seedlings were grown in LDs and harvested at ZT16. **a-c**, **e)** Values are means ± s.d. of three biological replicates. Two-tailed $t$ tests were conducted in (**a-c**) to evaluate statistically significant differences (*** $p$<0.001), while one-way ANOVA was conducted in (**e**), with letters to indicate statistically-distinct means ($p$<0.01).

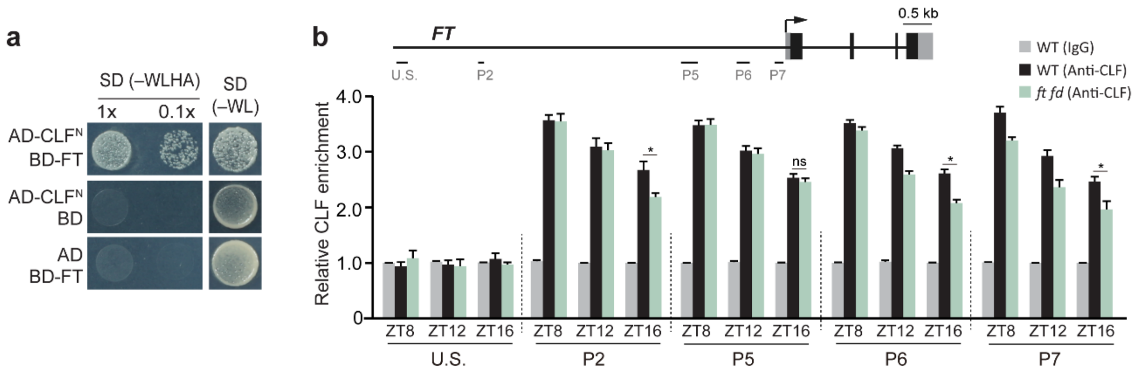


**Figure S6. FT interacts with CLF and is partly involved in CLF binding *FT* chromatin. a)** FT interacted with an N-terminal region of the CLF protein (aa 1 to 556) in yeast cells. Yeast was grown on a stringent selective medium lacking of tryptophan (W), leucine (L), histidine (H) and adenine (A), and cells grown on the SD media lacking of W and L serve as growth control. **b)** ChIP analysis of dynamic CLF enrichment on *FT* chromatin in WT and *ft fd* seedlings grown in LDs. Levels of *FT* fragments immunoprecipitated by rabbit polyclonal anti-CLF were quantified by qPCR and normalized to the internal control *TUB2*. Shown are relative fold enrichments of CLF in WT and *ft fd* over a background control (the immunoprecipitation with IgG). Values are means ± s.d. of three biological replicates. Statistical significance of indicated mean differences are assessed using two-tailed *t* test (* $p< 0.05$, and ns for not significant).

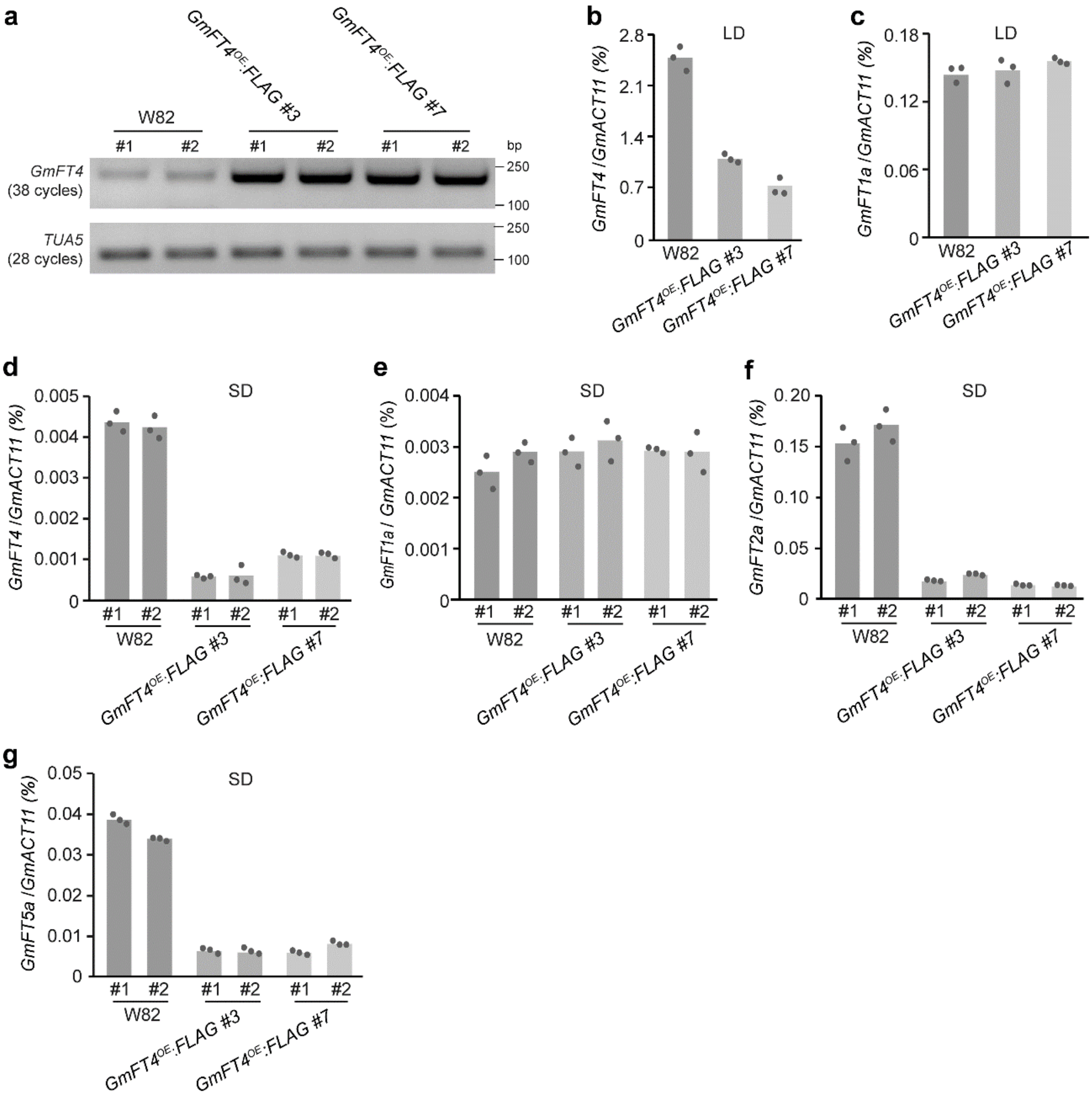


**Figure S7. Repression of *FT-like* genes by *GmFT4* overexpression in soybean. a)** Analysis of *GmFT4:FLAG* overexpression in leaves of the indicated transgenic soybean lines by semiquantitative RT-PCR. Two individual soybean plants from each transgenic line or *Williams 82* (grown in SDs) were examined. The constitutively-expressed *TUBULIN ALPHA5* (*TUA5*) serves as an internal control. W82 for *Williams 82*, the non-transgenic background. **b**,**c)** Transcript levels of endogenous *GmFT4* (**b**) and *GmFT1a* (**c**) in the leaves of W82 and *GmFT4$^{OE}$:FLAG* lines at 21 DAE under LDs. Leaves from two independent $T_3$ soybean lines (5 plants per line) were harvested at ZT16 for RNA extraction. Transcript levels were quantified by RT-qPCR and normalized to the constitutively expressed *GmACT11*. Values are means ± s.d. of three technical replicates. **d-g)** Transcript levels of endogenous *GmFT4* (**d**), *GmFT1a* (**e**)

*GmFT2a* (**f**), and *GmFT5a* (**g**), and in the leaves of W82 and *GmFT4*$^{OE}$*:FLAG* lines at 14 DAE under SDs. Samples were harvested at ZT8. Two individual soybean plants from each transgenic line or W82 were examined, and data points of three technical replicates for each sample are overlaid on bar graphs.

## Table S1. List of primers used in this study

| Experiment | Amplified region | Sequence (5'-3') |
|---|---|---|
| RT-PCR | *GmFT4* | F: CTACAGGGGCAACCACTGGAGAAGA |
| | | R: TCTCCTTCGTCCACCCCAACCA |
| | *GmTUA5* | F: TGCCACCATCAAGACTAAGAGG |
| | | R: ACCACCAGGAACAACAGAAGG |
| RT-qPCR | *FT* (CDS) | F: GACCTCAGGAACTTCTATACTTTGGTTATG |
| | | R: CTGTTTGCCTGCCAAGCTG |
| | *FT* (*3' UTR*) | F: ACTGGAACAACCTTTGGCAATGAGA |
| | | R: CAATTGGTTATAAAGGAAGAAGCCAT |
| | *FD* | F: GCAAGACTCAAGAGACAACAAG |
| | | R: CAAAATGGAGCTGTGGAAGAC |
| | *FT:HA* | F: TCCCACTGCAGGAATTCATCGT |
| | | R: TCGTATGGGTACTCGAGTGC |
| | *CO* | F: CACTACAACGACAATGGTTCC |
| | | R: GGTCAGGTTGTTGCTCTACTG |
| | *GUS* | F: CTCCTACCGTACCTCGCATTAC |
| | | R: ACGCGCTATCAGCTCTTTAATC |
| | *TUB2* | F: GCCTTGTACGATATTTGCTTCAGGAC |
| | | R: CGGAGGTCAGAGTTGAGTTGAC |
| | *UBQ10* | F: GATCTTTGCCGGAAAACAATTGGAGGA |
| | | R: CGACTTGTCATTAGAAAGAAAGAGATAAT |
| | *GmFT1a* | F: TGCAACTACAGGGGCAAACT |
| | | R: GTTGAAGTACATGGCCGCTA |
| | *GmFT2a* | F: GGATTGCCAGTTGCTGCTGT |
| | | R: GAGTGTGGGAGATTGCCAAT |
| | *GmFT5a* | F: GCCTTACTCCAGCTGATACT |
| | | R: GGCATGCTCTAGCATTGCAA |
| | *GmFT4* (*3 'UTR*) | F: CTACAGGGGCAACCACTGGAGAAGA |
| | | R: GGACAACGTGCGCAGTGTGA |
| | *GmACT11* | F: CGGTGGTTCTATCTTGGCATC |
| | | R: GTCTTTCGCTTCAATAACCCTA |
| ChIP-qPCR | *FT U.S.* | F: GCGGCATTGTACTAAACGAAA |
| | | R: GCCACCAAATCTTATCACCCT |
| | *FT P1* | F: ACGTAAATTATGACACTAAATGGTGC |
| | | R: GTGTTGACGTTGAGTTTGTGATCTT |

| | | |
|---|---|---|
| ChIP-qPCR | *FT P2* | F: ACGTTGATGATAGTGAAGTGA<br>R: ACGCAACCAAGTAGAGACGT |
| | *FT P2a* | F: CGTTGATGATAGTGAAGTGAGACATCTTGG<br>R: AAAACGTTTGGAAATAGGAAGTATGTAAAAACGAA |
| | *FT P3* | F: TGTAGCACAAGATCTTTTGGAGAACT<br>R: TGATTTCATTTTGTCGAGATTTAGAAG |
| | *FT P4* | F: ATCCACTTGCCAATCTTCGTAAT<br>R: TCATTGGTGTAATGACCATGATAAGA |
| | *FT P5* | F: GTGGCGGACAATCCATCTATCTC<br>R: GATCGACCATTGATAATCGAAATCGCA |
| | *FT P6* | F: GCAGATATCTTGTACTTAATTCATTTTG<br>R: GCCTTTTCTTGTAGTTATACCAGGTA |
| | *FT P7* | F: GTGTGGTGGGTTTGGAATACC<br>R: ACTCGGGTCGGTGAAATCAT |
| | *FT E1* | F: ATAGTAAGCAGAGTTGTTGGAGACG<br>R: CAATCTCAACTCTTGGCTTGTTTTG |
| | *FT I1* | F: GCCCCACGCTTTCCTTTTCTCTGTT<br>R: CACTTATGCAAGAAGTTGGTGGAAA |
| | *FT I2* | F: TCTCCATTGGTTTGTGCACTAACTC<br>R: TCGAAGTTGATTTTATATGTCTCCTTC |
| | *FT* ($P_7$)*-GUS* | F: CCAGTGTATTAGTGTGGTGGGT<br>R: GTTTTTTGATTTCACGGGTTGGGG |
| | *TUB2* | F: ATCCGTGAAGAGTACCCAGAT<br>R: AAGAACCATGCACTCATCAGC |
| | *GmFT2a P1* | F: ACTCCTTCTGATCTCAATTGTAAG<br>R: AACTAAATTTTTCTTATTGAGATTATC |
| | *GmFT2a P2* | F: CAACAAACAAGATGTATGGTTTTCA<br>R: TCATTCAATGGAATTATAAATCGTTT |
| | *GmFT2a P3* | F: TTCTAAAACAGAAAGAAACTCATTATAT<br>R: TAAACCTTTTACTTTGTGATTTAATA |
| | *GmFT2a P4* | F: GATAGATATTCGTTCACCATATAGAG<br>R: AATTAAGAATCATTGATTGACTTTC |
| | *GmFT2a P5* | F: ACTCAAGTGTTGCCAATTAATTGAC<br>R: CATCTTCTCATTTTCTCTCTTCCATT |
| | *GmFT2a I1* | F: CTCTTTTAACTTTGTATTCAAACAATC<br>R: GTACTTGACCTTCCCTTAAACACA |
| | *GmFT5a P1* | F: ATGTCCCTCATATTTGTAATAAAGCC<br>R: AATGATGATGCAAACACATTATTCAT |

| | | |
|---|---|---|
| ChIP-qPCR | *GmFT5a P2* | F: ATTAGGATTCCAACCAAACACAACT |
| | | R: AGATAAACCCTAAACTATTTTTGGAT |
| | *GmFT5a P3* | F: TGTAGATACCACGAAATAGAACTTACAT |
| | | R: AAAGAGATAATCTGTGTCCATATTCG |
| | *GmFT5a 3'UTR* | F: ATAATTTAGAGAGAAGGTACCATCTT |
| | | R: TAGCCAGAAAAGAAAGGACATCATAA |
| | *GmFT4 P1* | F: GATAAGTGTTTTAGTTGCCAAGAATG |
| | | R: GATATTGATAACTTCCATTGTCCATG |
| | *GmFT4 P2* | F: ATTTGTCTTGCTCTGTTTGTGCA |
| | | R: GTCCCTGAGTCCTGAATCCTAAAT |
| | *GmFT4 P3* | F: GTGGCTGTTGTAGAATAGAGGGA |
| | | R: AACTAAAGAACAAAATTAAAATAGCGA |
| | *GmFT4 P4* | F: AAGTCTCCTTGTTAAATTGCCTAATC |
| | | R: ATTAGAGCCTTTTCCTTTCTCATTG |
| | *GmFT4 P5* | F: AGTTCTGCCTCAAGTTTGTCTATAAAG |
| | | R: AGGCCTTCAAATATGACAAATCAC |
| | *GmFT4 P6* | F: GTCAAGGTGGCTCAATGAAGAAC |
| | | R: CACCACTAATCCAATTTTGATGG |
| | *GmFT4 P7* | F: TTTTGCATGACAAACACTAACCAC |
| | | R: GAAAAAGAGTCAGAAGAACGGGA |
| | *GmFT4 P8* | F: CTTAGAGGAAAAGGAAAACCAGAAT |
| | | R: GAGAAACAGAACTCGTGAAGGGA |
| | *GmACT11* | F: CGGTGGTTCTATCTTGGCATC |
| | | R: GTCTTTCGCTTCAATAACCCTA |
| 3C-qPCR | *FT I* | R: GACCATCCTGTGTTGACGTTGAGTTTG |
| | *FT II* | R: CTAATGTTGGCCAAGATGTCTCAC |
| | *FT III* | F: GGATTGACACATATCTCTTATATACTAGTG |
| | *FT anchor* | F: GAAGCAGAAACAAAAACAAGTAAAACAG |
| | *FT loading CK* | F: GGATTTTTCTTTGTTCCTCCTACCT |
| | | R: CTGGCTTGAATATCAGAATATCCTTAT |
